# Antiproliferative and Pro-apoptotic Effects of Sesquiterpene Lactones from *Schkuhria pinnata* in THP‑1 Leukemia Cells

**DOI:** 10.1021/acsomega.5c10243

**Published:** 2026-01-30

**Authors:** Dagmar Jankovská, Tereza Kauerová, Romana Kunovská, Martina Čudová, Jana Melicharová, Susanna Vogliardi, Karel Šmejkal, Peter Kollár, Milan Malaník

**Affiliations:** † Department of Natural Drugs, Faculty of Pharmacy, 37748 Masaryk University, Palackého 1946/1, 612 00 Brno, Czech Republic; ‡ Department of Pharmacology and Toxicology, Faculty of Pharmacy, Masaryk University, Palackého 1946/1, 612 00 Brno, Czech Republic; § Department of Pharmaceutical Sciences, Mass Spectrometry Facility, 9308 University of Padua, Via Francesco Marzolo 5, 35131 Padua, Italy

## Abstract

As *Schkuhria pinnata* is a food supplement commonly available on the European market, the aqueous-methanol extract from the aerial parts of *S. pinnata* was subjected to chromatographic separation to isolate compounds with antiproliferative and pro-apoptotic properties. This procedure led to the isolation of two previously undescribed sesquiterpene lactones (**1** and **2**) bearing an unusually modified germacranolide skeleton, four known heliangolides (**3**–**6**), together with flavonoids hispidulin and pectolinarigenin. The structures of isolated compounds were elucidated by a comprehensive analysis of HRMS and 1D and 2D NMR data. Sesquiterpene lactones (**1**–**6**) were evaluated for their antiproliferative activities in the THP-1, DU-145, and MCF-7 cancer cell lines. Compounds **3**–**6** showed an antiproliferative effect with the highest intensity in the THP-1 leukemic cell line. The most effective compound **5**, 2″-dehydroeucannabinolide semiacetal, induced apoptosis and loss of mitochondrial membrane potential, increased mitochondrial superoxide production, and suppressed mitochondrial ATP production in THP-1 cells.

## Graphical abstract

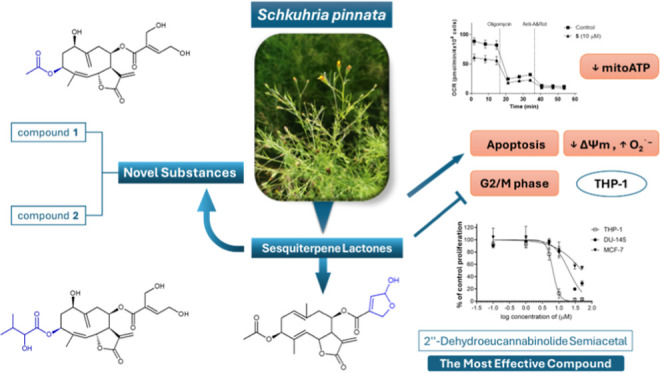

## Introduction

*Schkuhria pinnata* (Lam.) Kuntze ex Thell., Asteraceae, is an annual, inconspicuous herb native to South America, particularly the Andes region.^1^ However, this plant was also introduced to other countries, in particular, to the southern parts of Europe (Spain, France) and Africa (the Republic of South Africa, Uganda). The expansion of the cultivation of *S. pinnata* can be attributed to its medicinal properties. In South America, the decoction of branches and/or roots is traditionally used as an antiseptic and anti-inflammatory agent to treat allergies and skin-related disorders like dermatitis, acne, and eczema,^2^ while Ugandan healers used a decoction of leaves and flowers to manage pain and inflammation.^3^ There are also records about the usage of another *Schkuhria* species, *Schkuhria schkuhrioides* Thell., in Mexican ethnomedicine for the treatment of dermatological conditions with cancer symptomatology.^4^ Although the aerial parts of *S. pinnata* can be bought in health food stores or in medicinal plant shops throughout Europe under the name “canchalagua”, evidence of its safety and efficacy is scarce.

Recent scientific studies describe the extracts of aerial parts or leaves of *S. pinnata* as possessing promising antibacterial properties against strains causing acne,^5^ antinociceptive,^3^ antimycobacterial, and antioxidant activities^6^ and even potent glucose uptake ability.^7^ Moreover, *S. pinnata* acetone leaf extract displayed cytotoxic effects against the HeLa cancer cell line,^8^ while the antiproliferative activity of *S. pinnata* ethanol leaf extract was observed against the Caco-2 cancer cell line.^9^ Furthermore, an extract from the whole plant induced an antiproliferative effect in human hepatocarcinoma cell lines, where the highest potential was observed against Hep3B cells.^10^ However, all mentioned studies deal with various extracts, and thus, reported cytotoxic and antiproliferative activities of *S. pinnata* extracts have never been related to any particular compounds.

*S. pinnata* contains a variety of biologically active components, mainly sesquiterpene lactones,^11–14^ some acylated phenylpropanoids,^15,16^ flavonoids,^16,17^ and phytosterols.^18^ Some sesquiterpene lactones isolated from the aerial parts of *S. pinnata* have been described to exert significant antiprotozoal activity^13,19^ and moderate anti-inflammatory effects.^2,14^

However, the most pronounced bioactivities of sesquiterpene lactones are their antiproliferative and pro-apoptotic properties against cancer cells.^20–24^ Notably, artesunate, parthenolide, or thapsigargin derivatives were evaluated in cancer clinical trials.^22,25^ In general, sesquiterpene lactones bearing an α-methylene-γ-butyrolactone group are considered as promising anticancer agents due to their high reactivity that enables covalent interactions with biological targets.^25,26^ These compounds act as alkylating agents, forming stable adducts with nucleophilic sites in vivo, which leads to the inhibition of key enzymes and regulatory proteins.^21^ Sesquiterpene lactones exert anticancer effects also by modulating cellular redox balance and interfering with key signaling pathways, particularly NF-κB and STAT3 (signal transducer and activator of transcription 3), which are critical for cancer cell survival, proliferation, and resistance to therapy.^26^ Two of the most studied compounds, parthenolide and costunolide, belong to germacranolides, the same subgroup of sesquiterpene lactones as most of the constituents of *S. pinnata*. Although many studies describe antiproliferative and pro-apoptotic properties of parthenolide and costunolide,^20,24,26^ there is limited information about the anticancer potential of their derivatives present in *S. pinnata*. At first, hiyodorilactone A (eucannabinolide) and hiyodorilactone B displayed significant inhibitory activity against Ehrlich ascites carcinoma.^27^ Furthermore, eucannabinolide induced apoptosis in triple-negative breast cancer (TNBC) cells^28^ and the human breast cancer MCF-7 cell line.^29^ Eucannabinolide was even reported to induce antileukemic effect in the lymphocytic leukemia P 388 mouse model.^30^

As there are many reports in the literature describing sesquiterpene lactones as promising anticancer agents, our research is focused on the isolation of these compounds from aerial parts of *S. pinnata* and the evaluation of their antiproliferative activities in diverse cancer cell lines and their ability to trigger apoptosis.

## Results and Discussion

### Isolation and Structural Elucidation

Six sesquiterpene lactones (**1**–**6**) belonging to germacranolides were isolated from the aerial parts of *S. pinnata* together with flavonoids hispidulin and pectolinarigenin (Figure 1). Their structures were identified by NMR and HR-MS analyses and compared with the literature, as follows: schkuhrin I (syn. eucannabinolide) (**3**),^30^ schkuhrin II (**4**),^31^ 2″-dehydroeucannabinolide semiacetal (**5**),^32^ and hiyodorilactone B (**6**).^33^ Additionally, two flavonoids hispidulin and pectolinarigenin were identified as well,^34^ but these were not evaluated in biological assays due to their low yields.

**Figure 1:**
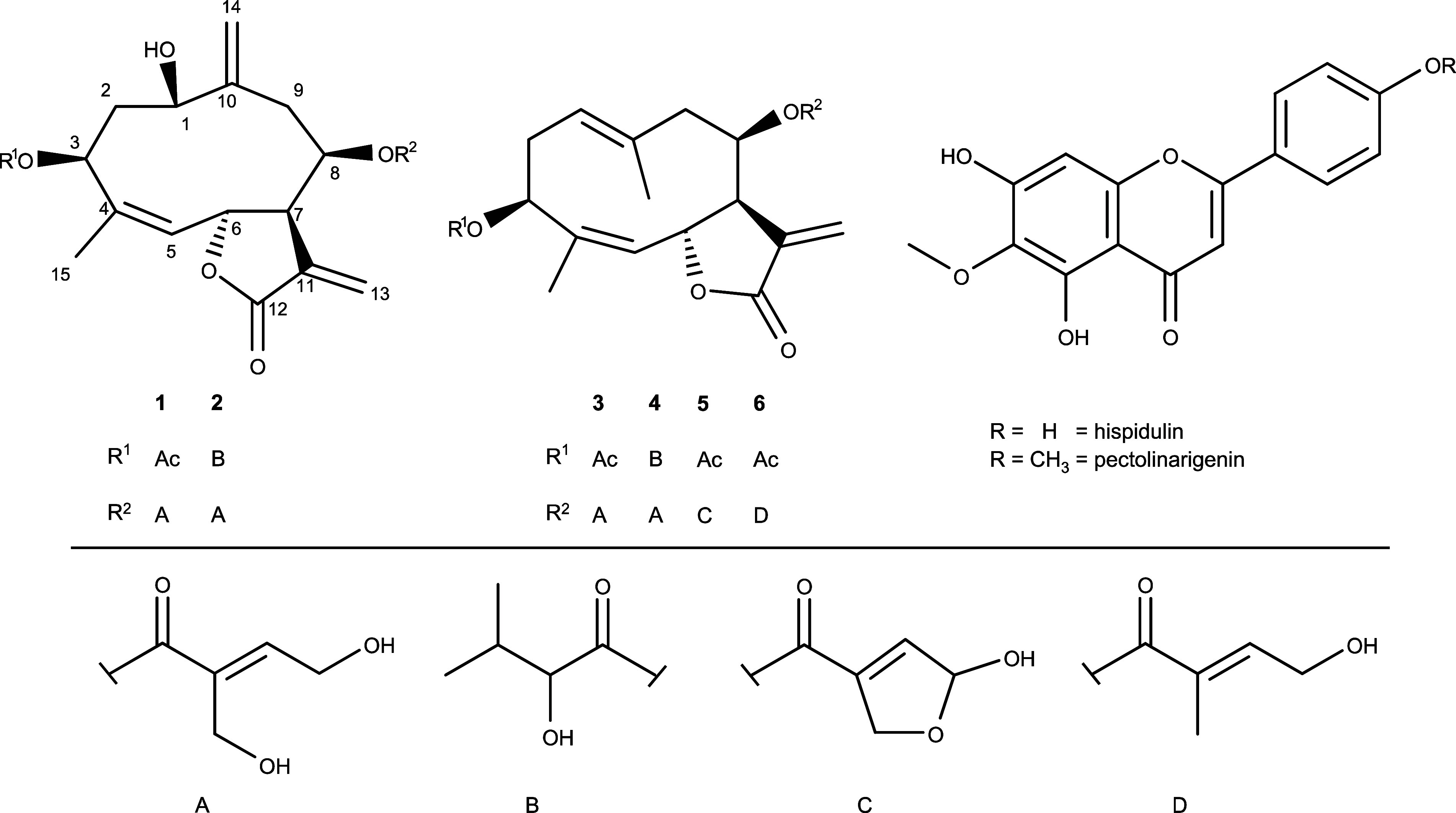
Structures of the compounds isolated from *S. pinnata*.

Compound **1** was isolated as a colorless oily substance. Its molecular formula was established as C_22_H_28_O_9_ based on the HRESIMS ion at *m*/*z* 459.1607 [M + Na]^+^ (calcd for C_22_H_28_O_9_Na, 459.1631). From ^1^H NMR data (Table 1), two methyl groups [δ_H_ 1.72 and 1.96 (each 3H, s)] and five olefinic protons [δ_H_ 4.98 (1H, br s), δ_H_ 5.21 (1H, br s), δ_H_ 5.27 (1H, dd, *J* = 1.7, 10.0 Hz), δ_H_ 5.98 (1H, d, *J* = 1.9 Hz), and δ_H_ 6.69 (1H, t, *J* = 5.6 Hz)] can be easily recognized. A combination of ^1^H NMR data with HSQC data revealed the presence of a CH group (δ_C_ 46.5/δ_H_ 3.22), two methylenes (δ_C_ 35.0/δ_H_ 1.98, 2.29; δ_C_ 37.0/δ_H_ 2.48, 2.62), two oxymethylenes (δ_C_ 55.7/δ_H_ 4.04; δ_C_ 58.3/δ_H_ 4.21), and finally four oxymethines (δ_C_ 69.7/δ_H_ 3.82; δ_C_ 76.7/δ_H_ 5.09; δ_C_ 73.7/δ_H_ 5.11; δ_C_ 73.9/δ_H_ 5.97). Based on a detailed evaluation of ^1^H–^1^H COSY and HMBC experiments (Figure 2), the germacranolide core was revealed together with acetyl and 4-hydroxy-2-(hydroxymethyl)-2-butenoyl groups. The position of the acetyl group was deduced based on the HMBC correlation of H-3 (δ_H_ 5.11) to C-1″ (δ_C_ 170.0). The configuration of the double bond at C-4 and C-5 is *Z* based on the NOESY correlation between H-5 (δ_H_ 5.27) with H_3_-15 (δ_H_ 1.72). As no NOESY correlation of H-3′ (δ_H_ 6.69) was observed, the comparison of the chemical shift of H-3′ (δ_H_ 6.69) with the literature data^35,36^ indicated that the 4-hydroxy-2-(hydroxymethyl)-2-butenoyl moiety must be 4′,5′-dihydroxytigloyl. This hypothesis was supported by the NOESY correlation between H-4′ (δ_H_ 4.21) and H-5′ (δ_H_ 4.04). Further NOESY correlations allowed us to establish complete stereochemistry, as depicted in Figure 3. All NMR data perfectly match those of hydroperoxyheterophyllin A^37^ with only one exception that, in compound **1**, the hydroperoxy group is exchanged with a simple hydroxy group. Accordingly, compound **1** was identified and named 1β-hydroxy-3β-acetoxy-8β-(4′,5′-dihydroxytigloyloxy)-germacra-*Z*4­(5),10­(14),11­(13)-trien-6α,12-olide.

**Table 1:** NMR Spectroscopic Data of Compounds **1** and **2** in DMSO-*d*_6_ (δ in ppm)

1			2	
position	δ_C_, type	δ_H_ (*J* in Hz)	δ_C_, type	δ_H_ (*J* in Hz)
1	69.7, CH	3.82, m	70.9, CH	3.84, m
2	35.0, CH_2_	α 2.29, t (13.8); β 1.98, q (3.0)	36.0, CH_2_	α 2.13, t (13.7); β 2.04, m
3	73.7, CH	5.11, dd (5.1, 2.3)	72.7, CH	5.26, dd (ov)
4	137.9, C		138.2, C	
5	126.5, CH	5.27, dd (10.1, 1.6)	126.2, CH	5.27, dd (ov)
6	73.9, CH	5.97, dd (10.1, 1.8)	74.2, CH	5.83, d (7.7)
7	46.5, CH	3.22, br s	45.5, CH	3.32, br s
8	76.7, CH	5.09, m	76.7, CH	5.18, m
9	37.0, CH_2_	α 2.62, dd (14.6, 5.3); β 2.48, dd (ov)	37.0, CH_2_	α 2.59, dd (14.9, 6.9); β 2.48, dd (ov)
10	143.6, C		144.0, C	
11	137.9, C		137.4, C	
12	169.7, C		169.7, C	
13	125.2, CH_2_	6.12, d (2.0); 5.98, d (2.0)	125.2, CH_2_	6.12, d (1.7); 5.99, d (1.7)
14	116.9, CH_2_	5.21, s; 4.98, s	116.7, CH_2_	5.17, s; 5.11, s
15	22.9, CH_3_	1.72, s	22.4, CH_3_	1.70, s
1′	165.6, C		166.0, C	
2′	131.0, C		131.2, C	
3′	146.9, CH	6.69, t (5.7)	146.3, CH	6.64, t (5.7)
4′	58.3, CH_2_	4.21, dd (9.8, 4.9)	58.1, CH_2_	4.21, t (5.4)
5′	55.7, CH_2_	4.04, d (5.6)	55.7, CH_2_	4.04, d (5.7)
1″	170.0, C		173.8, C	
2″	21.3, CH_3_	1.96, s	75.0, CH	3.80, dd (5.7, 4.6)
3″			31.8, CH	1.93, m
4″			19.4, CH_3_	0.89, d (6.8)
5″			17.2, CH_3_	0.80, d (6.8)
1–OH		4.85, d (6.6)		4.94, d (5.6)
4′–OH		4.96, d (ov)		4.98, t (5.4)
5′–OH		4.69, t (5.6)		4.67, t (5.7)

ov—signals overlapped; carbon chemical shifts were extracted from HSQC and HMBC data.

**Figure 2:**
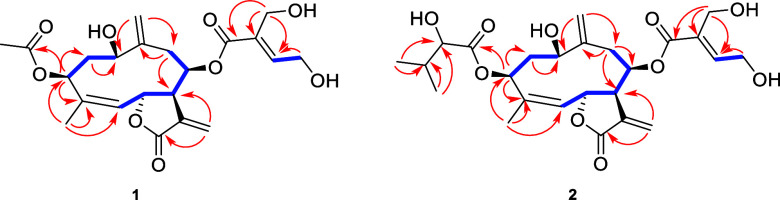
Key ^1^H–^1^H COSY (blue bold lines) and HMBC (red arrows) correlations of compounds **1** and **2**.

**Figure 3:**
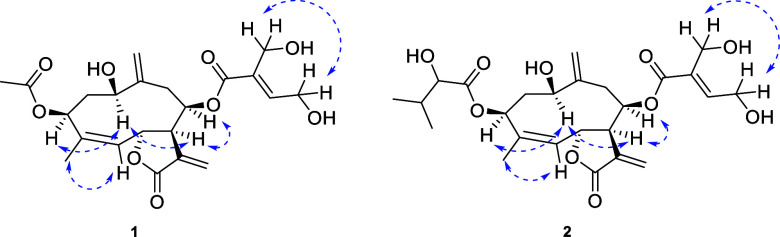
Key NOESY (blue dashed lines) of compounds **1** and **2**.

Compound **2** was isolated as a colorless oily substance with the molecular formula C_25_H_34_O_10_ based on the HRESIMS ion at *m*/*z* 517.2071 [M + Na]^+^ (calcd for C_25_H_34_O_10_Na, 517.2050). A detailed evaluation of ^1^H NMR, HSQC, ^1^H–^1^H COSY, and HMBC experiments (Table 1, Figure 2) revealed the presence of the same germacranolide-type sesquiterpene lactone skeleton with a 4′,5′-dihydroxytigloyl moiety at C-8 as in the case of compound **1**. The only difference is in the substituent at C-3 as remaining signals correspond to the 2″-hydroxyisovaleroyl moiety. Additionally, NOESY correlations (in CDCl_3_) (Figure 3) confirmed the same stereochemistry as that described above. Accordingly, compound **2** was identified and named 1β-hydroxy-3β-(2″-hydroxyisovaleroyloxy)-8β-(4′,5′-dihydroxytigloyloxy)-germacra-*Z*4­(5),10­(14),11­(13)-trien-6α,12-olide.

### Effect of Sesquiterpene Lactones 1–6 on the Proliferation of Human Cancer Cell Lines

Six sesquiterpene lactones extracted from *S. pinnata* were initially evaluated for their potential antiproliferative effect in three different human cancer cell lines: THP-1 monocytic leukemia cell line, DU-145 prostate cancer cell line, and the MCF-7 breast adenocarcinoma cell line. Among six tested compounds, **3**, **4**, **5**, and **6** induced a decrease in proliferation in all three cancer cell lines after 48 h of incubation, while the highest sensitivity toward their antiproliferative activity was observed in THP-1 cells (Table 2, Figure 4A). The IC_50_ value of the most potent compound **5** (6.67 μM) is comparable with that of parthenolide (4.7 μM).^38^ After the treatment of DU-145 cells with compound **5**, an approximately 3-fold higher IC_50_ value was obtained compared to the IC_50_ value in THP-1 cells. Similarly, in the case of compound **3** or **6**, approximately 1.8-fold and 1.4-fold higher IC_50_ values were obtained in DU-145 cells, respectively, compared to IC_50_ values in THP-1 cells. Concurrently, none of the tested compounds reduced the cell proliferation rate of MCF-7 cells below 50% compared with the control.

**Table 2:** Antiproliferative Effects of Tested Compounds **1**–**6** against Three Different Cancer Cell Lines

	IC_50_ (μM)^a^		
compound	THP-1^b^	DU-145^b^	MCF-7^b^
**1**	>50	>50	>50
**2**	>50	>50	>50
**3**	14.12 ± 2.25	25.55 ± 3.33	>50
**4**	36.05 ± 1.20	>50	>50
**5**	6.67 ± 0.78	19.92 ± 1.17	>50
**6**	14.94 ± 1.15	20.91 ± 4.63	>50

a IC_50_ values were determined using the WST-1 assay after 48 h of incubation of cells with various concentrations of the tested compounds. Mean value ± SD from three independent experiments, each performed in triplicate.

b THP-1: human monocytic leukemia cell line; DU-145: human prostate cancer cell line; MCF-7: human breast adenocarcinoma cell line.

Generally, the α-methylene-γ-butyrolactone group is considered to be responsible for the antiproliferative activity of sesquiterpene lactones.^25,26^ However, in our study, all tested compounds **1**–**6** contained this substituent, but two novel sesquiterpene lactones **1** and **2** did not suppress proliferation in any of the tested concentrations in all three cancer cell lines. This highlights the fact that orientation in space plays a crucial role, as well. The most studied sesquiterpene lactones, parthenolide and costunolide, possess a basic germacranolide skeleton, while compounds **1** and **2** contain another exocyclic methylene group that disrupts desirable orientation and thus diminishes the activity. However, distinct structural requirements are surely needed for the antiproliferative effect in different cancer cell lines.

To further examine which cell cycle phases are affected by the tested sesquiterpene lactones, we performed cell cycle analysis on THP-1 cells after their treatment with compounds **3**, **5**, and **6**, which previously manifested the highest antiproliferative potential. As shown in Figure 4B, out of the three tested compounds, compounds **3** and **5** induced a significant change in the distribution of cells in cell cycle phases. Both substances increased the number of cells in G2/M phases. Nevertheless, **5**, in correspondence with its previously described higher antiproliferative effect than **3**, also showed an even higher potential to affect cell cycle distribution when compound **5** concurrently reduced the percentage of cells in the G1/G0 phase. Carraz et al. showed that ethanolic extract from *S. pinnata* affected the course of the cell cycle of Hep3B cells in a similar manner.^10^ Even though the whole ethanolic extract was tested, Hep3B cells in the study also accumulated in the G2/M phase, while the number in the G1/G0 phase was decreased. Moreover, it was shown that cell cycle progression was blocked specifically in mitosis.^10^

Compound **5**, with the highest antiproliferative potential among the tested compounds against THP-1 cells, was also selected for further assessment of the difference in cytotoxicity toward the THP-1 cancer cell line and non-cancer primary cells, PBMCs (Figure 4C). While in THP-1 cells, compound **5** significantly reduced viability after 48 h of incubation already at a concentration of 5 μM, we observed only a slight reduction of viability in PBMCs at high concentrations of 30 μM or 50 μM. Such a difference of cytotoxicity toward cancer and non-cancer cells was also observed in the study published by Zhu et al.,^28^ who reported schkuhrin I (**3**) to induce cell death in TNBC human cancer cell lines, but nearly no cytotoxicity was detected against MCF-10A non-cancer mammary epithelial cells. Moreover, in the study by Carraz et al., the ethanolic extract from *S. pinnata* reduced the proliferation of cancer cells with higher intensity than the viability of non-cancer cells (IC_50_ = 9.5 μg/mL in Hep3B hepatocarcinoma cell line vs IC_50_ = 91.9 μg/mL in primary human hepatocytes).^10^

**Figure 4:**
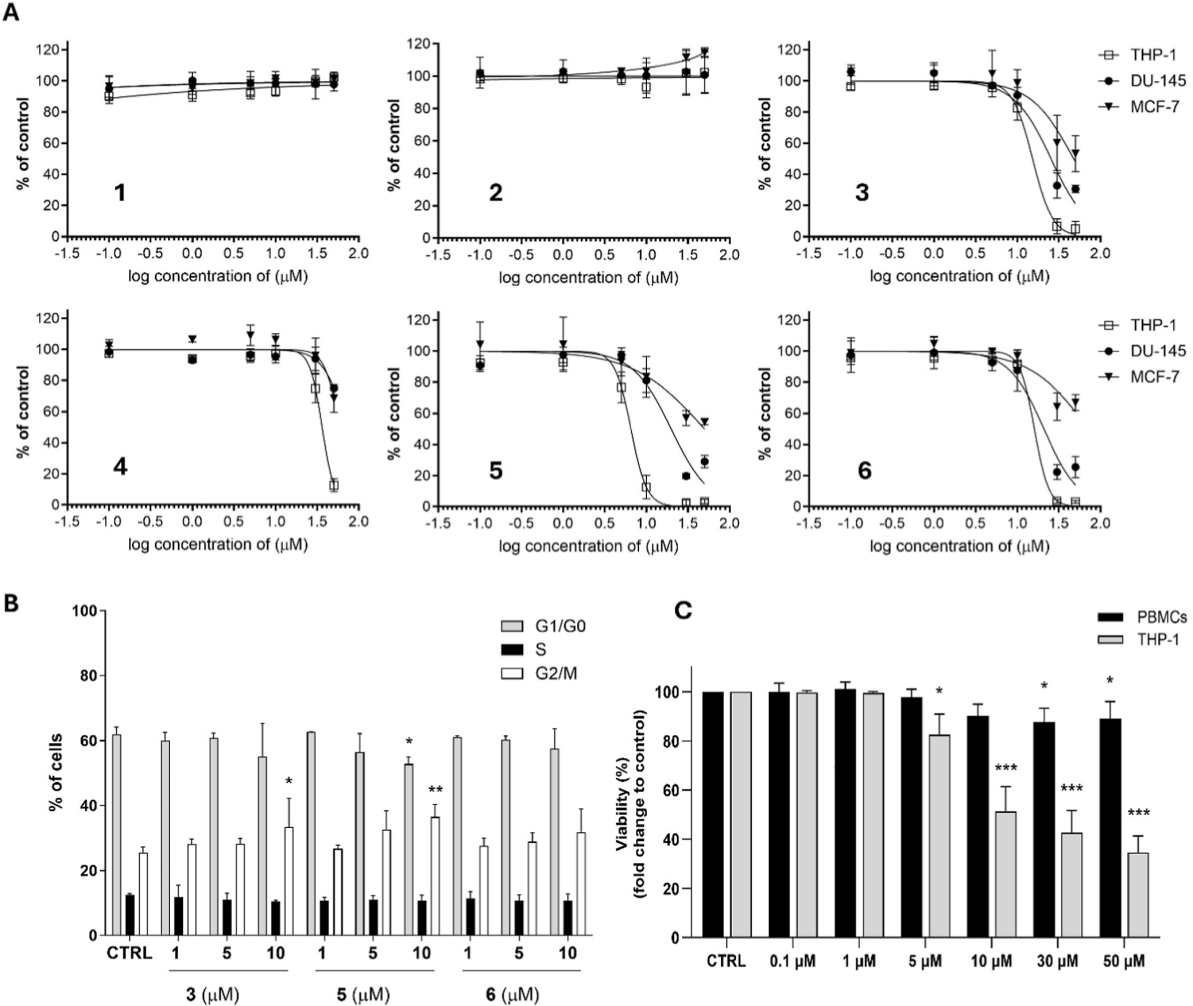
Effect of compounds **1**–**6** on proliferation and viability of human cancer cell lines and PBMCs. (A) Human cancer cell lines THP-1, DU-145, and MCF-7 were incubated with diverse concentrations of compounds **1**–**6** for 48 h, and subsequently, the rate of proliferation was determined by the WST-1 assay. The results are expressed as means ± SD from three independent experiments (*n* = 3), each performed in triplicate. (B) THP-1 cells were incubated with the indicated concentrations of compounds **3**, **5**, and **6** for 48 h. Subsequently, ethanol-fixed cells were stained with PI, and cell cycle analysis was performed using flow cytometry. The results shown are the means ± SD from three independent experiments (*n* = 3); *p < 0.05, **p < 0.01, significantly different from drug-free control (CTRL). (C) THP-1 monocytic leukemia cell line and PBMCs were incubated with diverse concentrations of compound **5** for 48 h, and cellular viability was assessed after PI staining using flow cytometry. The results shown are the means ± SD from three independent experiments (*n* = 3); *p < 0.05, ***p < 0.001, significantly different from drug-free control (CTRL).

### Sesquiterpene Lactones 3, 5, and 6 Exert Pro-apoptotic Effects in the THP-1 Monocytic Leukemia Cell Line

Schkuhrin I (**3**) was previously shown to induce apoptosis in different breast cancer cell lines, the MCF-7 cell line^29^ and two TNBC human cell lines MDA-MB-468 and MDA-MB-231.^28^ Since compounds **3**, **5**, and **6** in our study exert antiproliferative effects not only in the MCF-7 cell line, but much higher growth inhibitory potential was also observed in THP-1 cells, we aimed to examine their pro-apoptotic activity also in the monocytic leukemia cell line. As demonstrated in Figure 5A,B, our analysis that evaluates the presence of early and late stages of apoptosis revealed the highest pro-apoptotic potential in compound **5**. Even after 24 h of incubation, it intensively increases the number of cells in the early and, with less intensity, also in the late apoptotic stage, already at a concentration of 5 μM. Nonetheless, compounds **3** and **6** induced early apoptosis in THP-1 cells, namely, at a concentration of 10 μM, and compound **3** significantly increased the number of late apoptotic cells already at a concentration of 5 μM.

As all three compounds **3**, **5**, and **6** showed promising potential for triggering apoptosis in THP-1 cells in the Annexin V/PI assay, we aimed to further prove their pro-apoptotic potential by analyzing the presence of other selected apoptotic signs. Caspase 3 is a key protease responsible for the apoptosis progression. Caspase 3 occurs in the cell in an inactive form as a zymogen called procaspase. To ensure the progression of apoptosis, its activation is a crucial step. Once caspase 3 is activated, it can promote a cleavage of its cellular targets, such as poly­(ADP-ribose) polymerase (PARP), which is considered another marker of the ongoing process of apoptosis.^39–41^

In concert with the previous results of the Annexin V/PI assay, the highest pro-apoptotic potential of compound **5** was observed. This compound induced the most intensive activation of caspase 3 since a significant increase (*p* < 0.001) of the cell number with active caspase 3 was detected already at a concentration of 5 μM (Figure 5C,D). On the other hand, compounds **3** and **6** significantly activated caspase 3 only at a higher concentration of 10 μM. Additionally, all three tested compounds induced concentration-dependent cleavage of PARP (Figure 5E).

**Figure 5:**
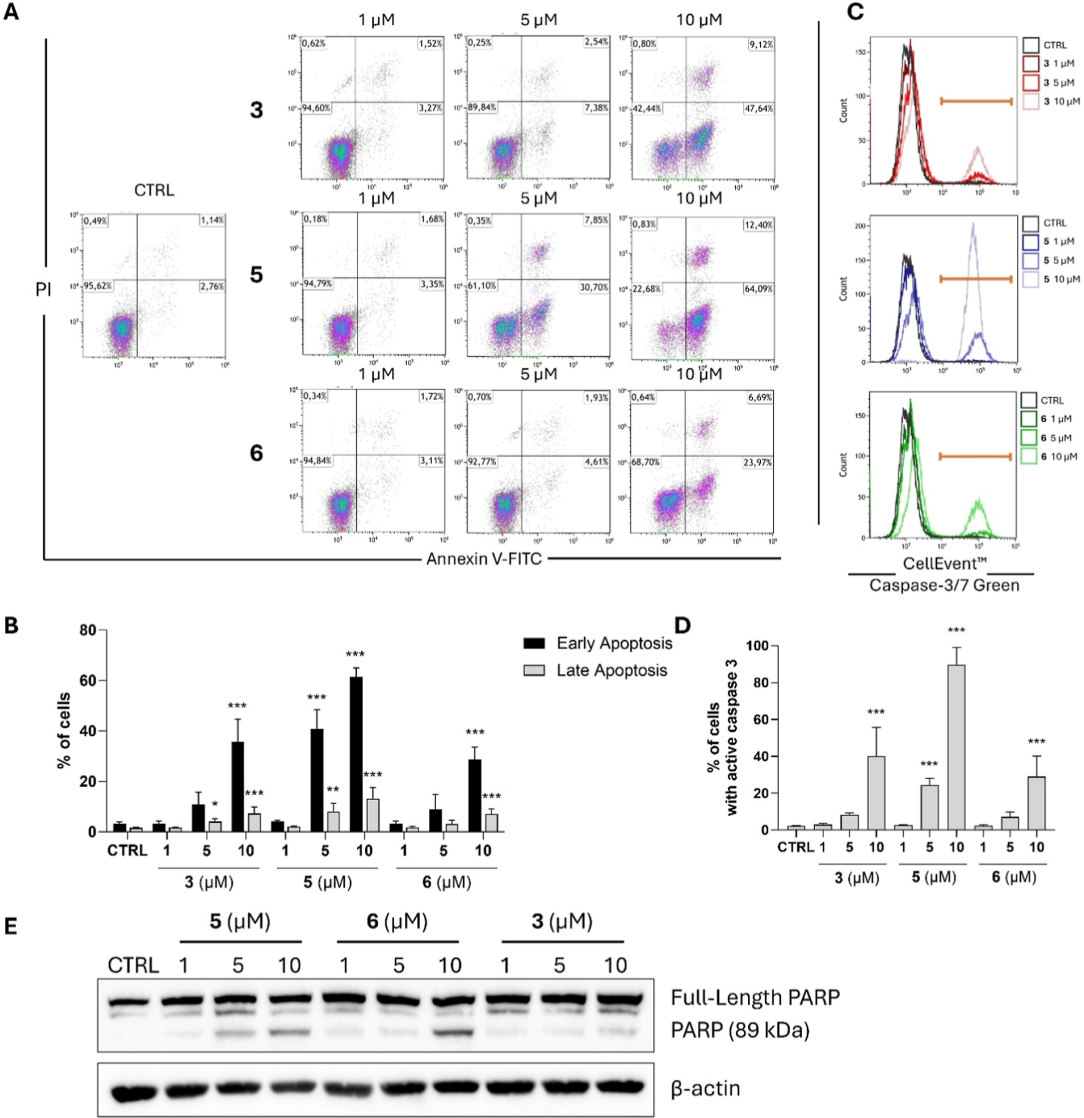
Compounds **3**, **5**, and **6** exerted pro-apoptotic effects in the THP-1 monocytic leukemia cell line. (A,B) THP-1 cells were treated with compounds **3**, **5**, and **6** and incubated for 24 h. The portion of cells undergoing early and late phases of apoptosis was determined by their staining with Annexin V-FITC and PI and measured by flow cytometry. (A) Representative dot plots are shown. (B) Results shown are the means ± SD from three independent experiments (*n* = 3); *p < 0.05, **p < 0.01, ***p < 0.001, significantly different from drug-free control (CTRL). (C,D) THP-1 cells were treated with compounds **3**, **5**, and **6** and incubated for 48 h. Subsequently, cells were stained by the CellEvent Caspase-3/7 Green reagent, and the frequency of cells with active caspase 3 was determined using flow cytometry. (C) Representative dot plots are shown. (D) Results shown are the means ± SD from three independent experiments (*n* = 3); ***p < 0.001, significantly different from drug-free control (CTRL). (E) THP-1 cells were treated with compounds **3**, **5**, and **6** and incubated for 48 h. The levels of full-length PARP protein and its cleaved form were detected by Western blotting. Representative immunoblots of one out of three experiments are shown. CTRL, drug-free control.

### Sesquiterpene Lactones 3, 5, and 6 Induce Mitochondrial Membrane Depolarization and Increase Production of Mitochondrial Superoxide

Previously, it was shown that the induction of apoptosis or necrosis in diverse cancer cells by the already mentioned germacranolides, parthenolide and costunolide, was related to alterations of mitochondrial functions. Namely, parthenolide dissipated mitochondrial membrane potential (ΔΨm) in stem-like cells derived from TNBC cell lines, and it also promoted ROS (reactive oxygen species) production.^42^ Parthenolide was further shown to provoke loss of mitochondrial membrane potential, leading to a leakage of cytochrome *C* and Smac pro-apoptotic proteins from mitochondria to cytosol in the COLO 205 colorectal adenocarcinoma cell line.^43^ Additionally, costunolide was reported to depolarize the mitochondrial membrane of gastric cancer cell line BGC82 ^44^ or of HL-60 leukemia cell line, where ROS levels were concurrently raised.^45^

In our analysis, all three sesquiterpene lactones **3**, **5**, and **6** showed the same ability to effectively dissipate ΔΨm and increase mitochondrial superoxide production in the THP-1 monocytic leukemia cell line (Figure 6A,B). The highest potential of ΔΨm dissipation was again observed in compound **5**, which dramatically elevated the number of cells with depolarized mitochondria to 69.0 ± 9.2% at a concentration of 10 μM versus 4.4 ± 0.8% in control (*n* = 3). Compounds **3** and **6** also decreased the mitochondrial membrane potential of the THP-1 cells. At a concentration of 10 μM, compound **3** elevated the percentage of cells with dissipation of ΔΨm to 35.8 ± 14.5%, and compound **6** to 26.8 ± 4.7% (*n* = 3). At the same time, compounds **3**, **5**, and **6** also intensified the production of mitochondrial superoxide (Figure 6C,D). All three compounds significantly elevated the levels of mitochondrial superoxide at a concentration of 10 μM, while compound **5** did so already at a concentration of 5 μM.

**Figure 6:**
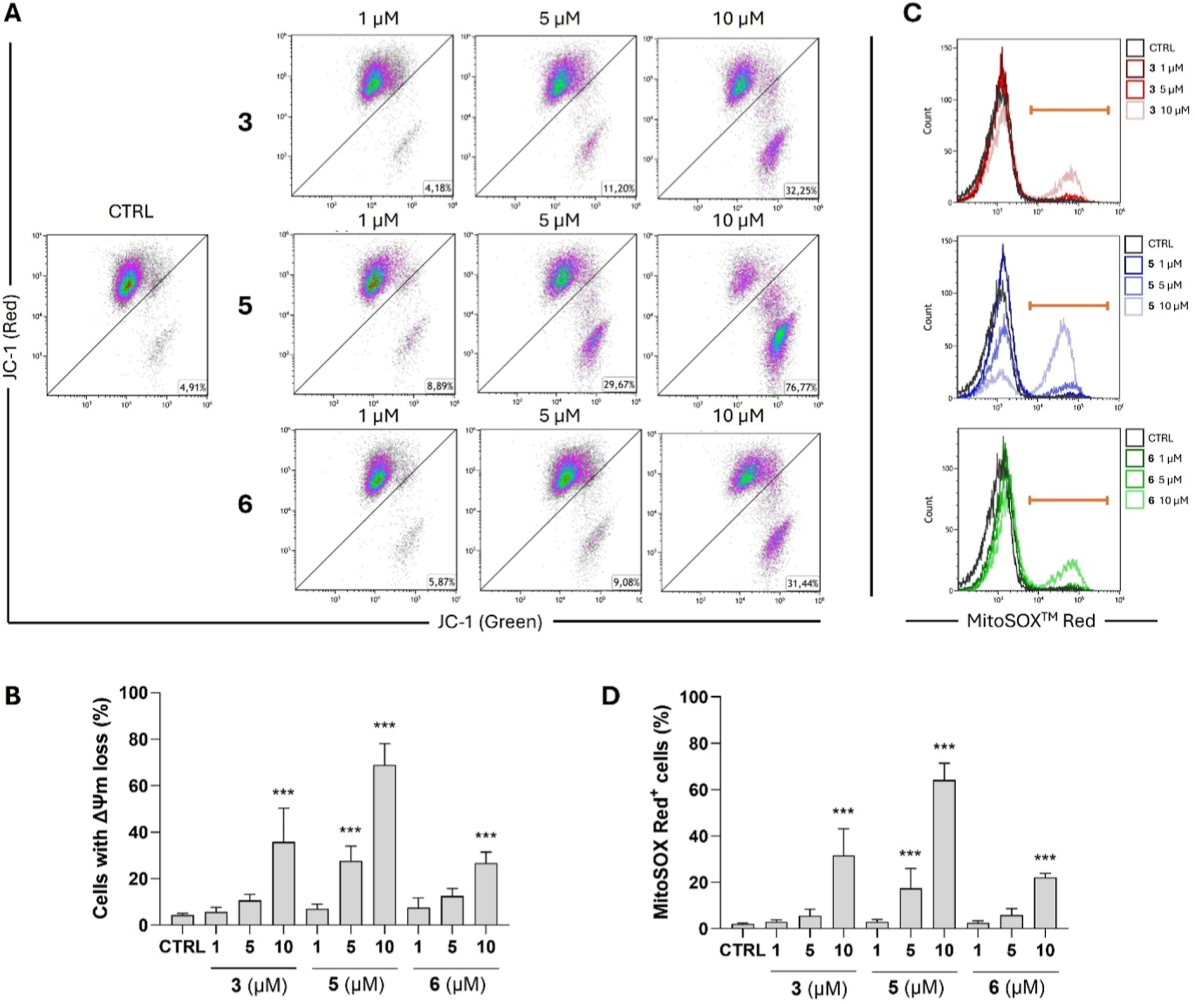
Compounds **3**, **5**, and **6** induced mitochondrial membrane depolarization and increased the production of mitochondrial superoxide in THP-1 cells. THP-1 cells were treated with compounds **3**, **5**, and **6** and incubated for 24 h. (A,B) The changes of the mitochondrial membrane potential (ΔΨm) were analyzed flow cytometrically after cellular staining with JC-1, a mitochondrial fluorescent probe. (A) Representative dot plots are shown. (B) Results shown are the means ± SD from three independent experiments (*n* = 3); ***p < 0.001, significantly different from drug-free control (CTRL). (C,D) The levels of mitochondrial superoxide were determined by flow cytometry after staining the cells with fluorescent probe MitoSOX Red. (C) Representative dot plots are shown. (D) Results shown are the means ± SD from three independent experiments (*n* = 3); ***p < 0.001, significantly different from drug-free control (CTRL).

### Compound 5 Impaired Mitochondrial ATP Production in the THP-1 Monocytic Leukemia Cell Line

In comparison with normal non-cancer cells, cancer cells exploit modified metabolic pathway patterns to ensure they meet their high energy requirements. Processes of cancer energetic metabolism are also adaptable, depending on the microenvironment and could possibly shift between two metabolic pathways: aerobic glycolysis and oxidative phosphorylation. Intensive research is currently underway in targeting metabolic processes in cancer cells with new anticancer agents, which could also potentially be helpful, e.g., in overcoming resistance to anticancer therapy.^46^

The ability to affect the energy metabolism of tumor cells was also described for the previously mentioned sesquiterpene lactone, parthenolide, which was observed to suppress oxidative phosphorylation (OXPHOS) in LCSCs (liver cancer stem cells) together with the ability to dissipate mitochondrial membrane potential and elevate ROS production.^47^ Given that our tested compounds **3**, **5**, and **6** also induced a loss of ΔΨm and potentiated the production of mitochondrial superoxide (a specific type of ROS in mitochondria), we decided to similarly assess the ability of the most potent compound **5** to impair the energetic metabolism of THP-1 cells. For the analysis, we used Seahorse technology, which enables us to distinguish the mitochondrial type of energetic metabolism from the glycolytic one.^48^ Our results showed a reduction in the oxygen consumption rate (OCR) (Figure 7A,B) and decreased the total ATP production rate (Figure 7C,D) by both tested concentrations of compound **5** (5 and 10 μM). The analysis further revealed different effects of compound **5** toward sources of ATP from mitochondrial respiration and glycolysis. The results showed decreased mitochondrial ATP production, while no significant change in glycolysis rate was observed (Figure 7E,F). As previously mentioned, targeting metabolic vulnerabilities in cancer cells is one of the current trends in antitumor drug development. The studied mechanisms of action also include inhibition of OXPHOS, and some already established inhibitors have even entered clinical trials.^49^ We can conclude that 2″-dehydroeucannabinolide semiacetal (**5**) induces growth inhibition and apoptosis in the THP-1 leukemia cell line while impairing mitochondrial functions and thus represents a promising compound for further preclinical studies in the field of anticancer drug development.

**Figure 7:**
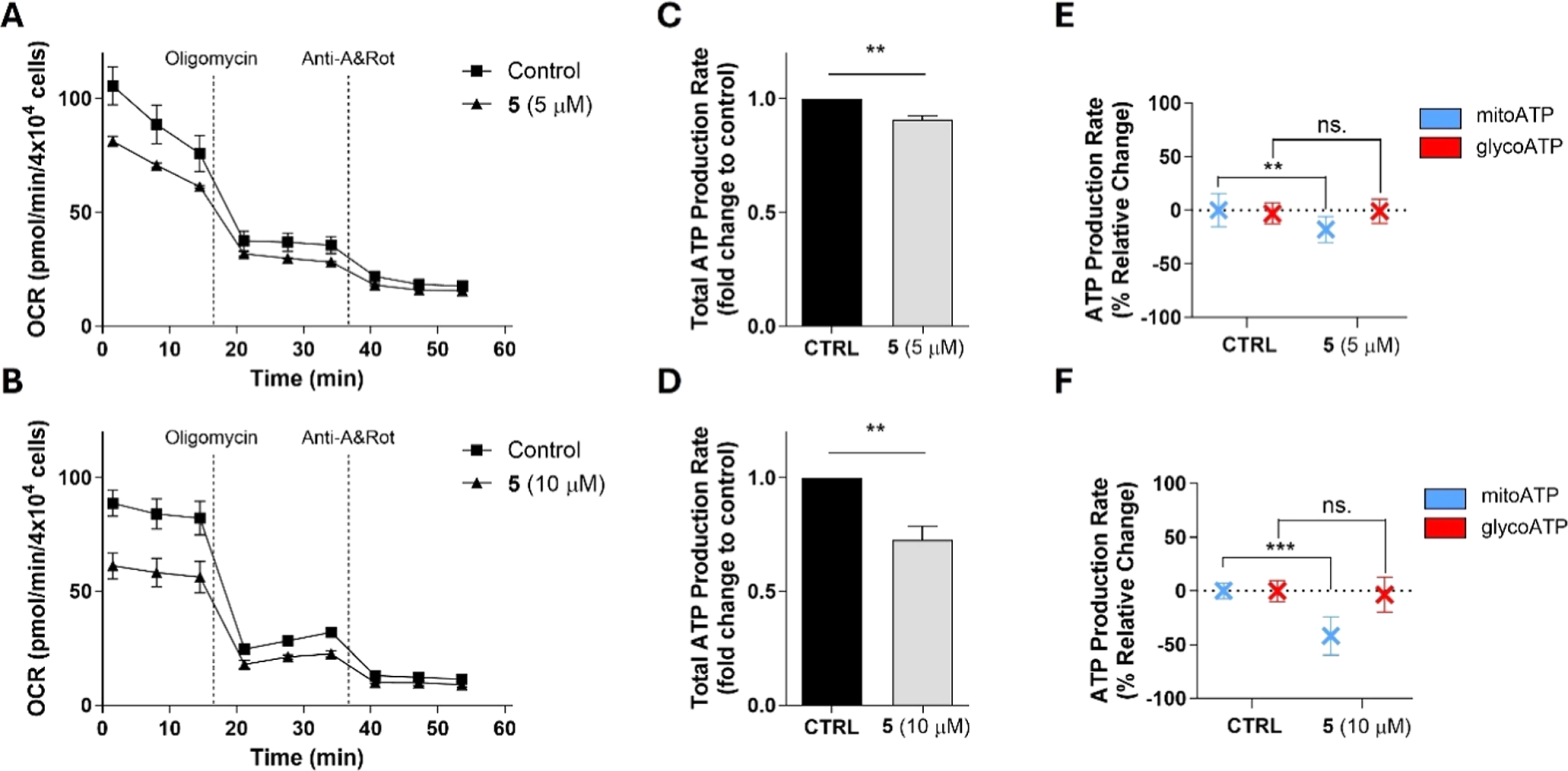
Compound **5** reduced mitochondrial ATP production in THP-1 cells. THP-1 cells were pretreated with (A) 5 μM or (B) 10 μM compound **5** for 5 h prior to the analysis. The ATP production rate was determined using the Agilent Seahorse XF Real-Time ATP Rate Assay Kit and the Seahorse XF HS Mini Analyzer. To assess the mitoATP and glycoATP production rate, oligomycin (1.5 μM) as an ATP synthase inhibitor and antimycin A with rotenone (Anti-A&Rot, 0.5 μM) as inhibitors of mitochondrial complexes III and I, respectively, were used to perform the analysis. (A,B) Representative results of changes in the oxygen consumption rate (OCR) induced by compound **5** are shown. Results of total ATP production rate (C,D) and mitoATP and glycoATP production rate (E,F) are shown as means ± SD from three independent experiments (*n* = 3); **p < 0.01; ***p < 0.001, significantly different from drug-free control (CTRL).

## Conclusion

The aerial parts of *S. pinnata* are commonly traded on the European market as a medicinal plant. However, information regarding this species remains scarce. In the present study, we isolated compounds from plant material belonging to the sesquiterpene lactone group, specifically four known and two previously undescribed sesquiterpene lactones of the heliangolide type (compounds **1** and **2**). To the best of our knowledge, sesquiterpene lactones bearing a germacranolide skeleton with two exocyclic methylene groups (**1** and **2**) were isolated from *S. pinnata* for the first time, and this study therefore enriched the known compounds of *S. pinnata* with another type. Sesquiterpene lactones represent a noteworthy class of natural compounds with a wide range of biological activities. However, the most pronounced bioactivities of the sesquiterpene lactone group are the antiproliferative and pro-apoptotic properties against diverse cancer cells. In our study, out of six sesquiterpene lactones (**1**–**6**) isolated from *S. pinnata*, 2″-dehydroeucannabinolide semiacetal (compound **5**) showed the highest potential to induce antiproliferative effects in the THP-1 monocytic leukemia cell line. Concurrently, compound **5** promoted apoptosis, caused a loss of mitochondrial membrane potential, elevated production of mitochondrial superoxide production, and reduced mitochondrial ATP production. Thus, our study demonstrates the potential of 2″-dehydroeucannabinolide semiacetal (**5**) for further investigation into its anticancer properties.

## Experimental Section

### General Experimental Procedures

The UV spectra were obtained using an Agilent 1100 chromatographic system with a DAD (Agilent Technologies, Santa Clara, CA, USA). ECD spectra were recorded on a JASCO J-815 CD spectrometer (Jasco, Easton, MD, USA). Semi-preparative HPLC was performed on a Dionex Ultimate 3000 system equipped with a UV detector (Thermo Fisher Scientific, Waltham, MA, USA) with an Ascentis RP-Amide column, 25 cm × 10 mm, 5 μm (Supelco, Bellefonte, PA, USA). Direct-injection HRMS analyses were performed using a Xevo G2-S Q-Tof instrument (Waters, Milford, Massachusetts, USA). Mass spectra were acquired in the 50–2200 *m*/*z* range using the following parameters: capillary 1.5 kV, source temperature 100 °C, desolvation temperature 350 °C. 1D and 2D NMR spectra were obtained on a JEOL ECZR 400 MHz NMR spectrometer (JEOL, Tokyo, Japan). Gradient-grade MeCN for HPLC was purchased from Sigma-Aldrich, USA, and other analytical-grade solvents were sourced from Lach-Ner, Czech Republic.

### Plant Material

The aerial parts of *S. pinnata* used in this study were collected in September 2021 at the Medicinal Herbs Centre of Faculty of Medicine, Masaryk University, Brno (49°12′03″N 16°35′04″E; alt.: 280 m a.s.l.), and identified by Ing. Anna Novotná. A voucher specimen (ID: SP2021) has been deposited in the Herbarium of the Department of Natural Drugs, Masaryk University, Brno.

### Extraction and Isolation

The air-dried aerial parts of *S. pinnata* (86.2 g) were crushed before being extracted three times with 90% methanol. After evaporation and lyophilization, 11.6 g of crude extract was obtained and then successively partitioned with *n*-hexane and ethyl acetate (EtOAc). The EtOAc-soluble fraction (3.347 g) was subjected to silica gel column chromatography (75 cm × 4 cm) using a mobile phase consisting of *n*-hexane/EtOAc 6:4 (*v/v*) to afford 42 subfractions (1–42). After that, the mobile phase was changed to *n*-hexane/EtOAc 4:6 (*v/v*) to afford 13 subfractions (43–55), and then changed to *n*-hexane/EtOAc 2:8 (*v/v*) to afford another 16 subfractions (56–71). Separation of subfraction 70–71 (400 mg) was performed by semi-preparative HPLC. The flow rate was 5 mL/min, UV detection was applied at 210, 254, 280, and 350 nm, and an injection volume of 35 μL was injected several times until the entire sample was completely consumed. The mobile phase consisted of 20% of MeCN (A) and 80% of H_2_O (B), with the gradient over 23 min reaching a composition of 50% A and 50% B. This procedure led to the isolation of four compounds: **1** (15.3 mg, *t*_*R*_ 6.8 min), **2** (6.1 mg, *t*_*R*_ 8.7 min), **3** (29.9 mg, *t*_*R*_ 15.6 min), and **4** (7.9 mg, *t*_*R*_ 18.6 min). Combined subfractions 43–47 (141.5 mg) were purified by semi-preparative HPLC (25%–55% MeCN, 23 min) to obtain compounds **5** (4.1 mg, *t*_*R*_ 15.3 min) and **6** (3.6 mg, *t*_*R*_ 17.8 min). Combined subfractions 32–42 (21.6 mg) were subjected to semi-preparative HPLC using a mobile phase consisting of 30% of MeCN (A) and 70% of H_2_O and 0.2% HCOOH (B), with the gradient over 20 min reaching a composition of 75% A and 25% B, yielding hispidulin (1.9 mg, *t*_*R*_ 13.5 min) and pectolinarigenin (1.3 mg, *t*_*R*_ 16.3 min).

1β-Hydroxy-3β-acetoxy-8β-(4′,5′-dihydroxytigloyloxy)-germacra-*Z*4­(5),10­(14),11­(13)-trien-6α,12-olide (**1**). Colorless oily substance; UV (DMSO) λ_max_ 224 nm; ECD (c 0.1, MeOH) λ_max_ (Δε) 214 (−0.05) nm; ^1^H and ^13^C NMR data, see Table 1; HRESIMS *m*/*z* 459.1607 [M + Na]^+^ (calcd for C_22_H_28_O_9_Na, 459.1631).

1β-Hydroxy-3β-(2″-hydroxyisovaleroyloxy)-8β-(4′,5′-dihydroxytigloyloxy)-germacra-*Z*4­(5),10­(14),11­(13)-trien-6α,12-olide (**2**). Colorless oily substance; UV (DMSO) λ_max_ 225 nm; ECD (c 0.1, MeOH) λ_max_ (Δε) 215 (−0.11) nm; ^1^H and ^13^C NMR data, see Table 1; HRESIMS *m*/*z* 517.2071 [M + Na]^+^ (calcd for C_25_H_34_O_10_Na, 517.2050).

### Cell Cultures, Conditions, and Reagents

All cancer cell lines used in this study, THP-1, DU-145, and MCF-7, were obtained from the American Type Culture Collection (ATCC, Manassas, VA, USA). THP-1 and DU-145 cells were maintained in RPMI-1640 medium, while MCF-7 cells were cultured in DMEM. All media were supplemented with 10% fetal bovine serum (Capricorn Scientific, Ebsdorfergrund, Germany) and an antibiotic solution containing 100 U/mL penicillin and 100 μg/mL streptomycin (Biosera, Cholet, France). PBMCs (peripheral blood mononuclear cells) acquired from healthy donors were purchased frozen from Lonza (Basel, Switzerland) and were maintained in RPMI-1640 medium supplemented with 10% human serum (Capricorn Scientific, Ebsdorfergrund, Germany) and 100 U/mL penicillin and 100 μg/mL streptomycin (Biosera, Cholet, France). Cells were incubated at 37 °C in a water-saturated atmosphere with 5% CO_2_. Dimethyl sulfoxide (DMSO) (Sigma-Aldrich, St. Louis, MO, USA) was used to dissolve the tested compounds, and the final concentration of DMSO did not exceed 0.1% (*v/v*) in any assay.

### Analysis of Cell Proliferation and Cytotoxicity

DU-145 cells, MCF-7 cells, and THP-1 cells were seeded in 96-well plates and incubated with tested compounds (concentrations ranging from 0.1 to 50 μM) for 48 h (37 °C, 5% CO_2_). Then, cell proliferation was evaluated using Cell Proliferation Reagent WST-1 [2-(4-iodophenyl)-3-(4-nitrophenyl)-5-(2,4-disulfophenyl)-2H-tetrazolium] (Roche Diagnostics, Mannheim, Germany), as was described previously.^50,51^ The IC_50_ values were calculated with GraphPad Prism 5.03 software (GraphPad Software, San Diego, CA, USA). Cytotoxicity analysis was performed using THP-1 cells, and the PBMCs treated with compound **5** at concentrations ranging from 0.1 to 50 μM. Cytotoxicity was assessed after 48 h incubation (37 °C, 5% CO_2_), whereas cells were harvested, washed with 1× PBS, and stained with the propidium iodide (PI) reagent at a final concentration of 20 μg/mL. After 15 min of incubation at room temperature protected from light, cytotoxicity was evaluated using a BriCyte E6 flow cytometer (Mindray, Shenzhen, China) in the PE channel (laser: 488 nm). Data evaluation was performed using Kaluza Flow Cytometry Analysis Software 2.1 (Beckman Coulter).

### Cell Cycle Analysis

THP-1 cells were treated with the tested compounds for 48 h at final concentrations ranging from 1 to 10 μM. Following the treatment, the cells were fixed in ethanol and subsequently stained with propidium iodide (PI) as described previously.^52^ DNA content analysis was performed by flow cytometry, with at least 1 × 10^4^ cells analyzed per sample. The percentage of cells in distinct cell cycle phases was calculated using Kaluza Analysis Software 2.1 (Beckman Coulter, Brea, CA, USA).

### Analysis of Pro-apoptotic Potential by Flow Cytometry

At the end of incubation with tested compounds at concentrations of 1, 5, and 10 μM, THP-1 cells were harvested and washed with 1× PBS. The incubation time for the Annexin V-FITC/PI assay was 24 h, and it was 48 h for the Caspase 3 activation assay. For the Annexin V-FITC/PI assay, THP-1 cells were stained according to the manufacturer’s protocol with an eBioscience Annexin V-FITC Apoptosis Detection Kit (Invitrogen, Thermo Fisher Scientific, Waltham, MA, USA). THP-1 cells were resuspended in 100 μL of 1× binding buffer, and Annexin V-FITC reagent [final concentration 1.5% (*v/v*)] and PI [final concentration 10% (*v/v*)] were added to each sample. For the Caspase 3 activation assay, THP-1 cells were resuspended in 1× PBS + 5% FBS solution with the reagent from the CellEvent Caspase-3/7 Green Detection Reagent kit (Invitrogen, Thermo Fisher Scientific, Waltham, MA, USA), at a final concentration of 2 μM. Samples were incubated at 37 °C without CO_2_ for 15 min in the case of the Annexin V assay or 30 min in the case of the Caspase 3 activation assay. The analysis was performed by flow cytometry. The intensity of fluorescence was determined in the FITC or PE channel (laser: λ 488 nm). Data evaluation was performed using Kaluza Flow Cytometry Analysis Software 2.1 (Beckman Coulter).

### Western Blotting

After 48 h of incubation of THP-1 cells with tested compounds at concentrations of 1, 5, and 10 μM, cell lysates were prepared, and protein concentration was adjusted. Next, SDS-PAGE with Western blotting was carried out according to the previously described procedure.^52^ Subsequently, membranes were incubated with the specific primary antibody overnight at 4 °C: *anti*-β-actin (catalog no. sc-47778), purchased from Santa Cruz Biotechnology (Santa Cruz, CA, USA), and *anti*-PARP (catalog no. 9542), purchased from Cell Signaling Technology (Danvers, MA, USA). Membranes were then stained with the appropriate secondary antibody: antimouse IgG, HRP-linked antibody (Cat. #: 7076) and antirabbit IgG, HRP-linked antibody (Cat. #: 7074) purchased from Cell Signaling Technology. Detection of proteins was performed with an Amersham ECL Prime Western Blotting Detection Reagent (Cytiva, Marlborough, MA, USA). The assessment of band intensity was performed with ImageJ software (National Institute of Mental Health, Bethesda, MD, USA).

### Evaluation of Mitochondrial Membrane Potential and Superoxide Production

THP-1 cells were incubated for 24 h with the tested compounds at concentrations ranging from 1 to 10 μM. To assess mitochondrial membrane potential, cells were stained with the fluorescent probe JC-1 (Invitrogen, Thermo Fisher Scientific, MA, USA) in 1× PBS at a final concentration of 10 μg/mL for 30 min at 37 °C and protected from light. Green (FITC) and red (PE) fluorescence intensities were measured using flow cytometry (488 nm laser). To evaluate mitochondrial superoxide levels, THP-1 cells were stained with a MitoSOX Red Superoxide indicator (Invitrogen, Thermo Fisher Scientific, MA, USA) in 1× PBS at a final concentration of 500 nM for 30 min at 37 °C, protected from light. Flow cytometry analysis was performed on the PerCP channel (488 nm laser). Data analysis was conducted using Kaluza Flow Cytometry Analysis Software version 2.1 (Beckman Coulter).

### Quantification of ATP Production from Glycolysis and OXPHOS

THP-1 cells were pretreated with compound **5** at concentrations of 5 or 10 μM for 5 h prior to the analysis. For the analysis, cells were seeded into PDL-coated tissue culture plates at a density of 4 × 10^4^ per well to achieve optimal confluence and centrifuged at 1300 rpm for 1 min to ensure adherence. The cells were then kept in the XF Real-Time ATP Rate Assay RPMI Medium (pH 7.4) supplemented with 10 mM glucose, 1 mM pyruvate, and 2 mM l-glutamine (Agilent Technologies, Santa Clara, CA) in a non-CO_2_ incubator prior to measurement for a maximum of 1 h. According to the manufacturer’s protocol (Agilent Technologies, Santa Clara, CA), the sensor cartridge was hydrated overnight in ddH_2_O and then calibrated in the calibrant solution for at least 1 h. Cartridge ports were loaded with oligomycin (15 μM, port A) and a mixture of rotenone and antimycin A (5 μM, port B), achieving final concentrations of 1.5 μM (oligomycin) or 0.5 μM (rotenone and antimycin A) in the well after their injection. The oxygen consumption rate (OCR) and extracellular acidification rate (ECAR) were measured using a Seahorse XF HS Mini Analyzer (Agilent Technologies, Santa Clara, CA). Even the seeding density in the wells was checked by counting the cells using a hemocytometer.

### Statistical Analysis

Experimental data are expressed as the mean ± the standard deviation (SD). Statistical significance was evaluated by one-way analysis of variance (ANOVA) paired with Dunnett’s post hoc test or by a paired *t*-test using the GraphPad Prism 5.03 software (GraphPad Software, San Diego, CA, USA).

## Supplementary Material



## Notes

Spectroscopic data for compounds **1–6** (HRMS and 1D and 2D NMR) (PDF)
