# Supplementary material for: Antiproliferative and Pro-apoptotic Effects of Sesquiterpene Lactones from Schkuhria pinnata in THP‑1 Leukemia Cells
Source: ACS Omega. 2026 Jan 30;11(6):9774–84. doi: 10.1021/acsomega.5c10243 (PMC12917833; doi:10.1021/acsomega.5c10243)
Supplement: Supplementary file 1 [file ao5c10243_si_001.pdf]

Supporting information

Antiproliferative and Pro-Apoptotic Effects of Sesquiterpene Lactones from *Schkuhria pinnata* in THP-1 leukemia cells

Dagmar Jankovská [a§], Tereza Kauerová [b§], Romana Kunovská [a], Martina Čudová [b], Jana Melicharová [b], Susanna Vogliardi [c], Karel Šmejkal [a], Peter Kollár [b*], Milan Malaník [a*]

[a] Department of Natural Drugs, Faculty of Pharmacy, Masaryk University, Palackého 1946/1, 612 00 Brno, Czech Republic

[b] Department of Pharmacology and Toxicology, Faculty of Pharmacy, Masaryk University, Palackého 1946/1, 612 00 Brno, Czech Republic

[c] Department of Pharmaceutical Sciences, Mass Spectrometry Facility, University of Padua, Via Francesco Marzolo 5, 35131 Padua, Italy

* Email: malanikm@pharm.muni.cz

* Email: kollarp@pharm.muni.cz

§ D.J. and T.K. contributed equally to this work and share first authorship.

Table of Contents

Figure S1. HRMS spectrum of compound **1** in positive mode
Figure S2. ^{1}H NMR spectrum of compound **1** in DMSO-d_6 in the range of 0–4.0 ppm
Figure S3. ^{1}H NMR spectrum of compound **1** in DMSO-d_6 in the range of 4.0–8.0 ppm
Figure S4. APT spectrum of compound **1** in DMSO-d_6
Figure S5. HSQC spectrum of compound **1** in DMSO-d_6
Figure S6. HMBC spectrum of compound **1** in DMSO-d_6
Figure S7. COSY spectrum of compound **1** in DMSO-d_6
Figure S8. NOESY spectrum of compound **1** in DMSO-d_6
Figure S9. HRMS spectrum of compound **2** in positive mode
Figure S10. ^{1}H NMR spectrum of compound **2** in DMSO-d_6 in the range of 0–4.0 ppm
Figure S11. ^{1}H NMR spectrum of compound **2** in DMSO-d_6 in the range of 4.0–8.0 ppm
Figure S12. APT spectrum of compound **2** in DMSO-d_6
Figure S13. HSQC spectrum of compound **2** in DMSO-d_6
Figure S14. HMBC spectrum of compound **2** in DMSO-d_6
Figure S15. COSY spectrum of compound **2** in DMSO-d_6
Figure S16. NOESY spectrum of compound **2** in $CDCl_3$
Figure S17. HRMS spectrum of compound **3** in positive mode
Figure S18. ^{1}H NMR spectrum of compound **3** in $CDCl_3$ in the range of 0–4.0 ppm
Figure S19. ^{1}H NMR spectrum of compound **3** in $CDCl_3$ in the range of 4.0–8.0 ppm
Figure S20. APT spectrum of compound **3** in $CDCl_3$
Figure S21. HSQC spectrum of compound **3** in $CDCl_3$
Figure S22. HMBC spectrum of compound **3** in $CDCl_3$
Figure S23. COSY spectrum of compound **3** in $CDCl_3$
Figure S24. NOESY spectrum of compound **3** in $CDCl_3$
Figure S25. HRMS spectrum of compound **4** in positive mode
Figure S26. ^{1}H NMR spectrum of compound **4** in $CDCl_3$ in the range of 0–4.0 ppm
Figure S27. ^{1}H NMR spectrum of compound **4** in $CDCl_3$ in the range of 4.0–8.0 ppm
Figure S28. APT spectrum of compound **4** in $CDCl_3$
Figure S29. HSQC spectrum of compound **4** in $CDCl_3$
Figure S30. HMBC spectrum of compound **4** in $CDCl_3$
Figure S31. COSY spectrum of compound **4** in $CDCl_3$
Figure S32. NOESY spectrum of compound **4** in $CDCl_3$
Figure S33. HRMS spectrum of compound **5** in positive mode
Figure S34. ^{1}H NMR spectrum of compound **5** in DMSO-d_6 in the range of 0–4.0 ppm
Figure S35. ^{1}H NMR spectrum of compound **5** in DMSO-d_6 in the range of 4.0–8.0 ppm
Figure S36. APT spectrum of compound **5** in DMSO-d_6
Figure S37. HSQC spectrum of compound **5** in DMSO-d_6

Figure S38. HMBC spectrum of compound **5** in DMSO-d_6
Figure S39. COSY spectrum of compound **5** in DMSO-d_6
Figure S40. NOESY spectrum of compound **5** in DMSO-d_6
Figure S41. HRMS spectrum of compound **6** in positive mode
Figure S42. ^{1}H NMR spectrum of compound **6** in DMSO-d_6 in the range of 0–4.0 ppm
Figure S43. ^{1}H NMR spectrum of compound **6** in DMSO-d_6 in the range of 4.0–8.0 ppm
Figure S44. APT spectrum of compound **6** in DMSO-d_6
Figure S45. HSQC spectrum of compound **6** in DMSO-d_6
Figure S46. HMBC spectrum of compound **6** in DMSO-d_6
Figure S47. COSY spectrum of compound **6** in DMSO-d_6
Figure S48. NOESY spectrum of compound **6** in DMSO-d_6

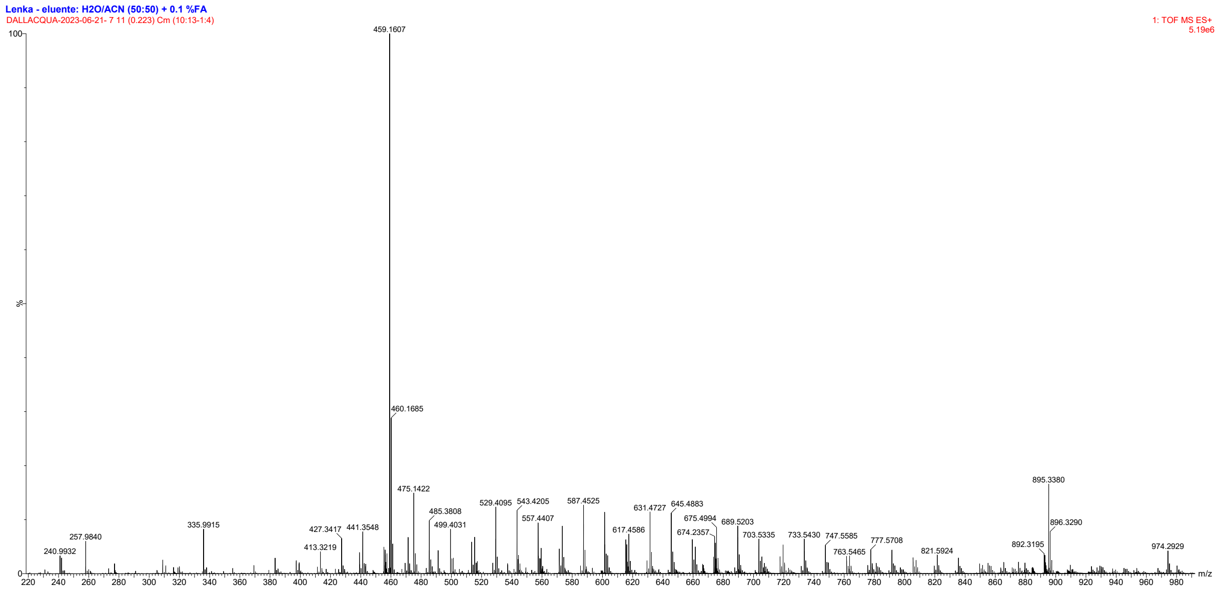

Figure S1. HRMS spectrum of compound **1** in positive mode

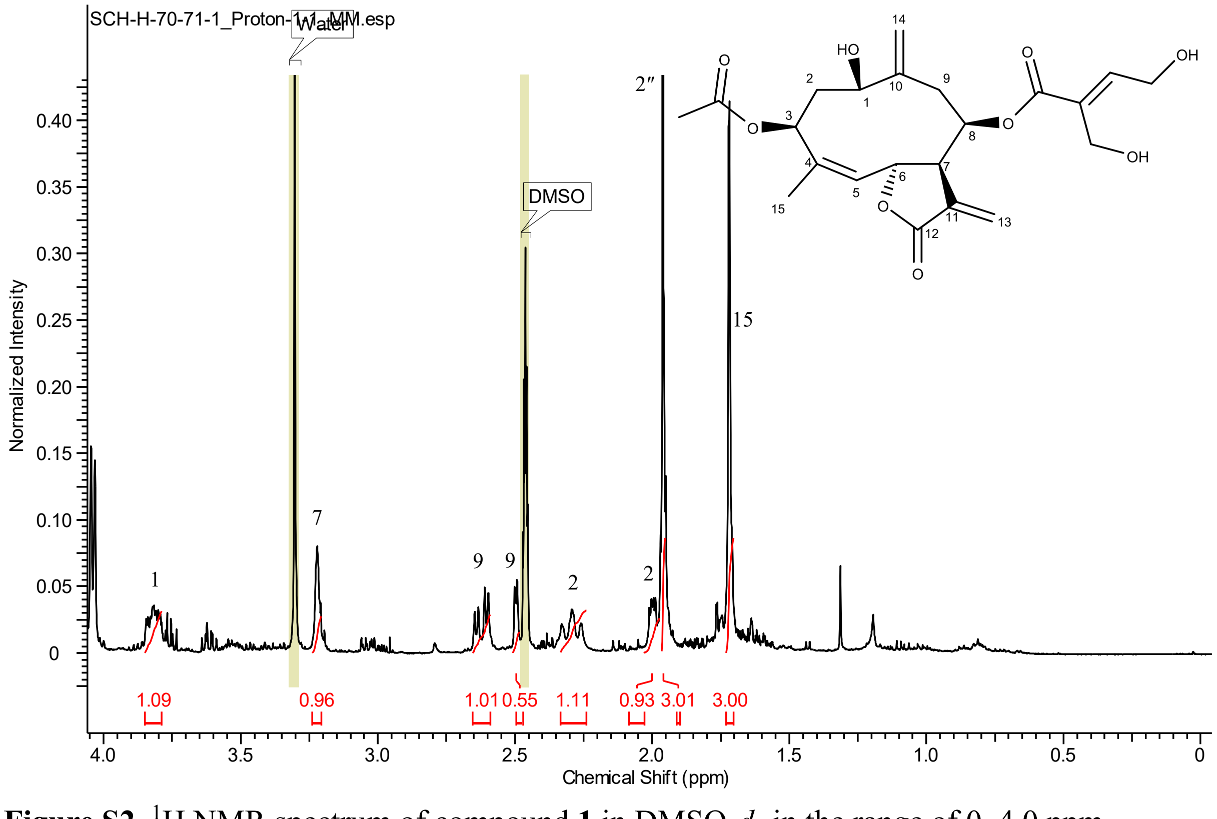

Figure S2. ^{1}H NMR spectrum of compound **1** in DMSO-d_6 in the range of 0–4.0 ppm

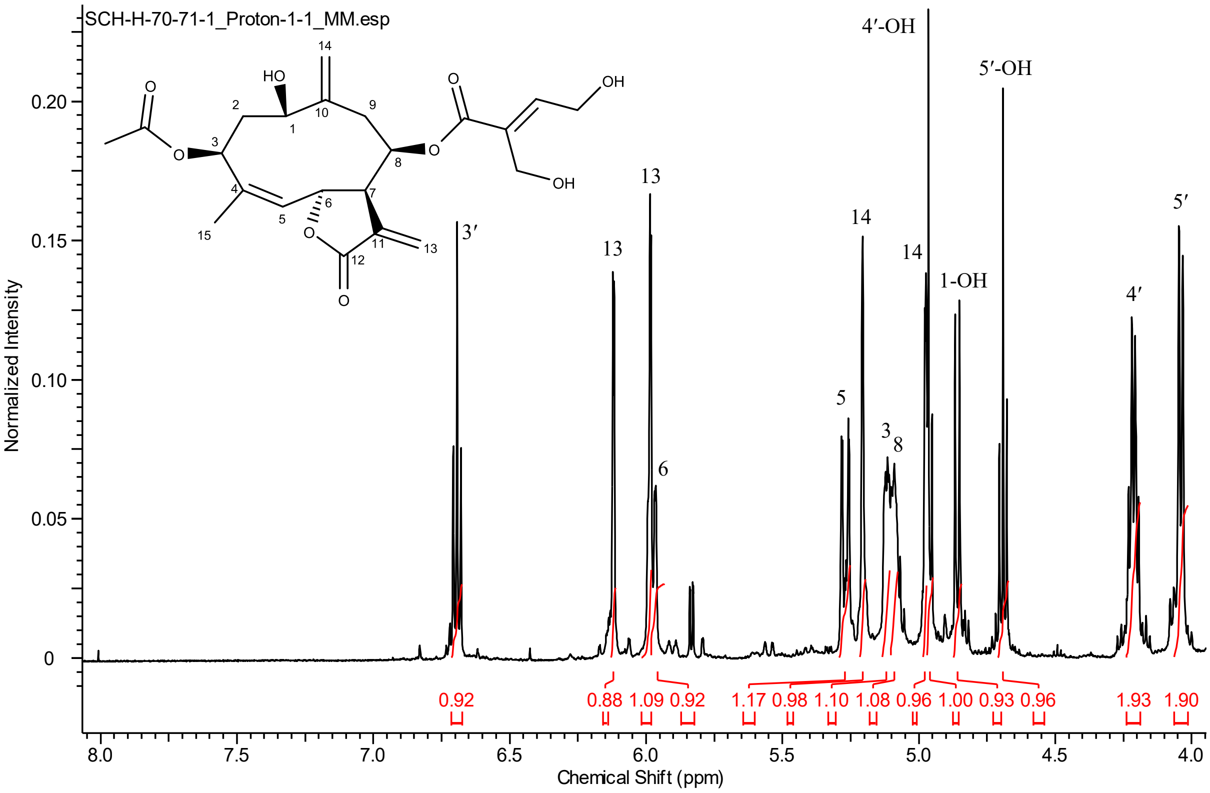

Figure S3. ¹H NMR spectrum of compound **1** in DMSO-d_6 in the range of 4.0–8.0 ppm

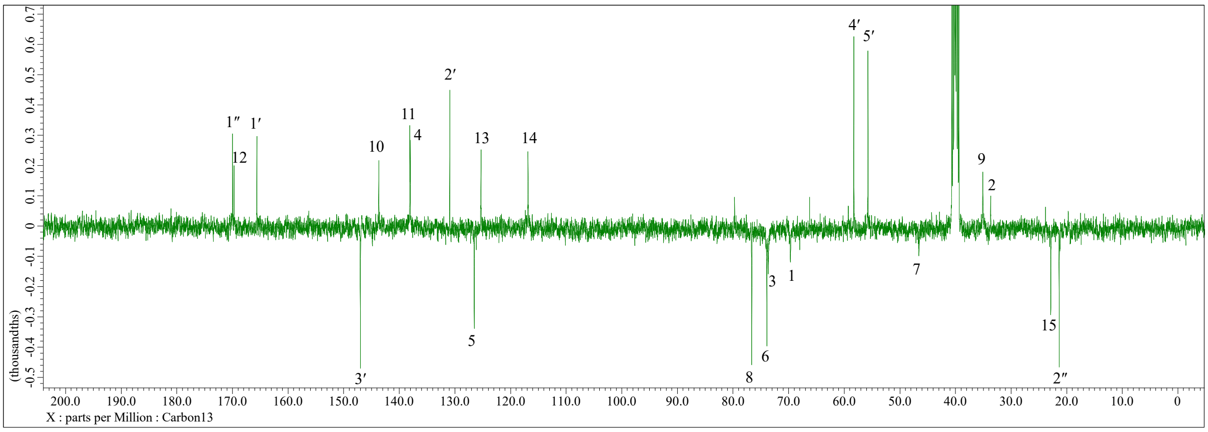

Figure S4. APT spectrum of compound **1** in DMSO-d_6

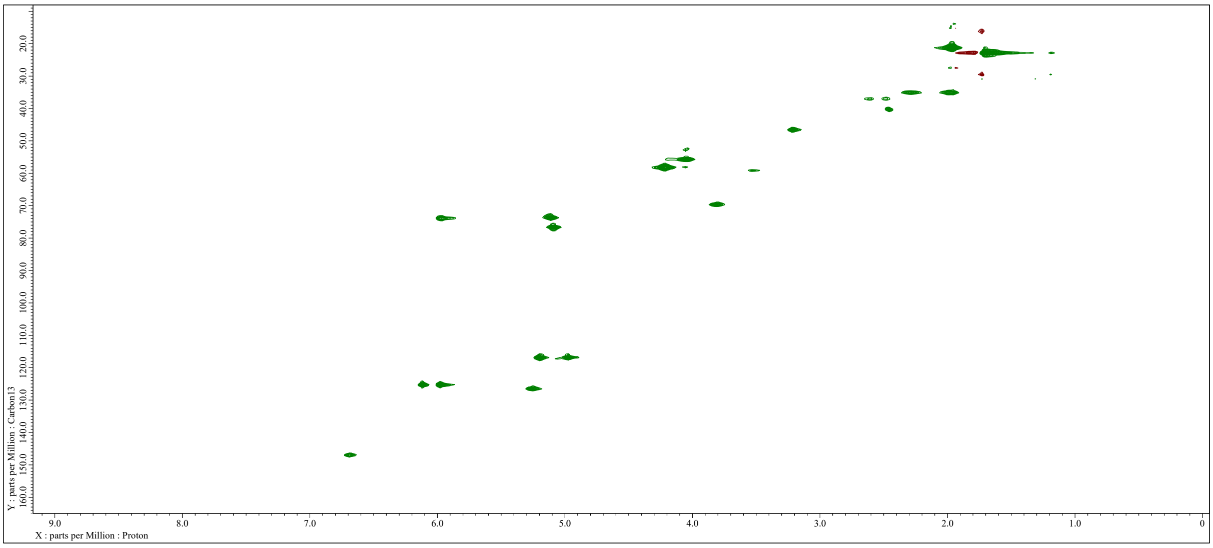

Figure S5. HSQC spectrum of compound **1** in DMSO-d_6

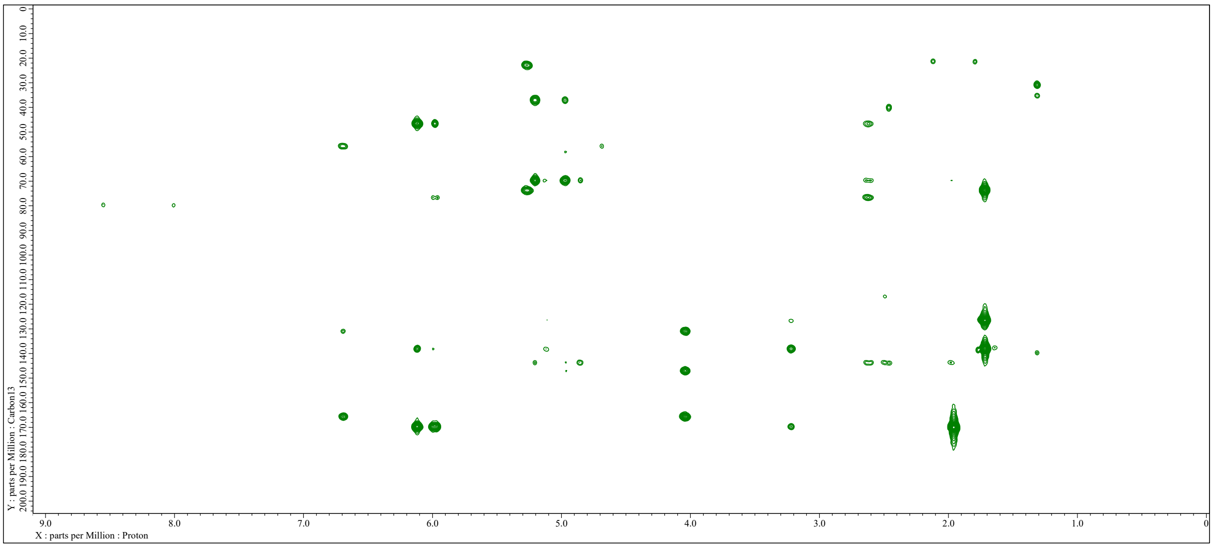

Figure S6. HMBC spectrum of compound **1** in DMSO-d_6

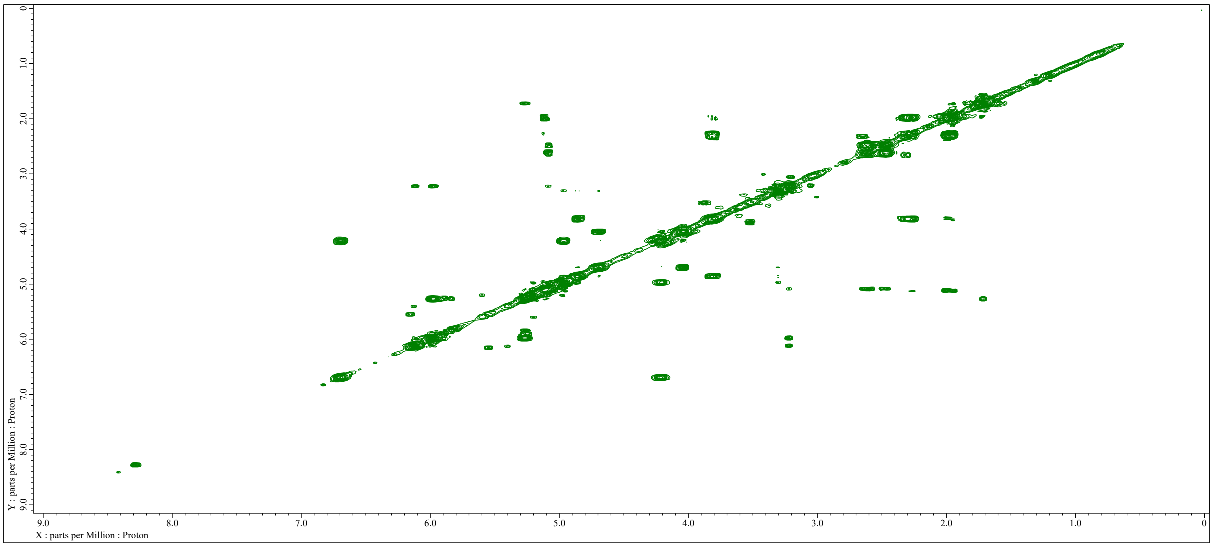

Figure S7. COSY spectrum of compound **1** in DMSO-d_6

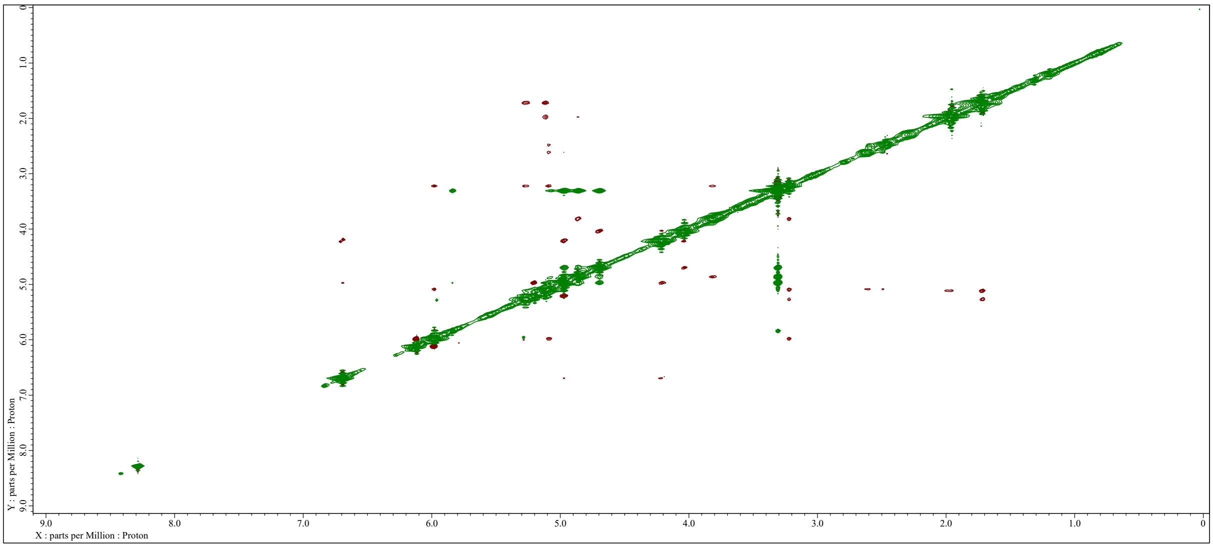

Figure S8. NOESY spectrum of compound **1** in DMSO-d_6

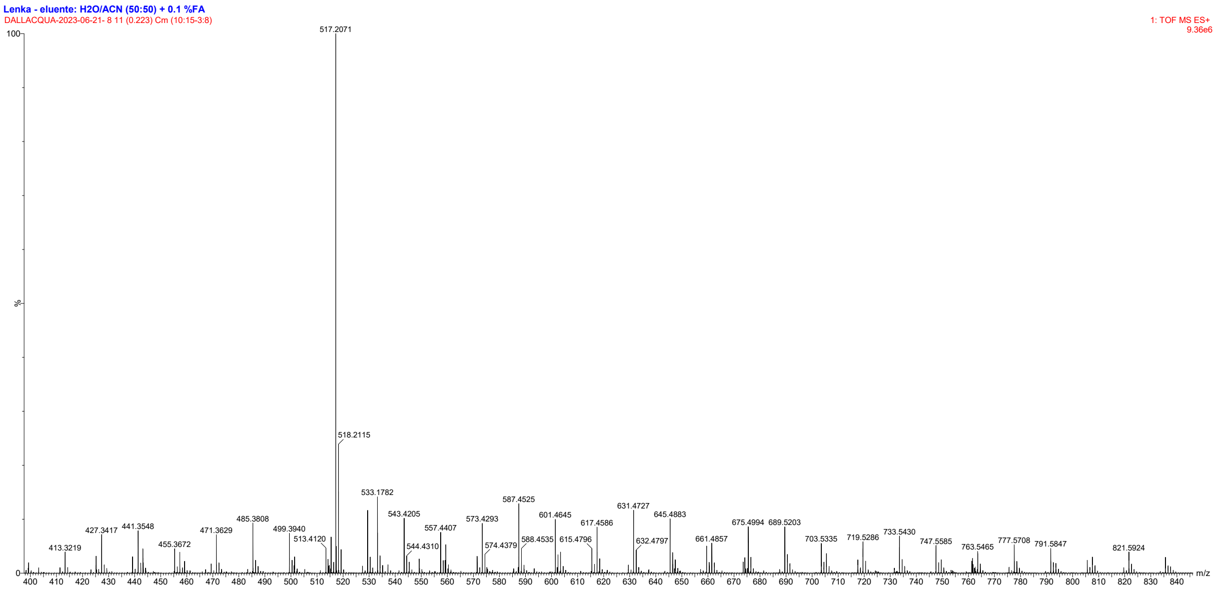

Figure S9. HRMS spectrum of compound **2** in positive mode

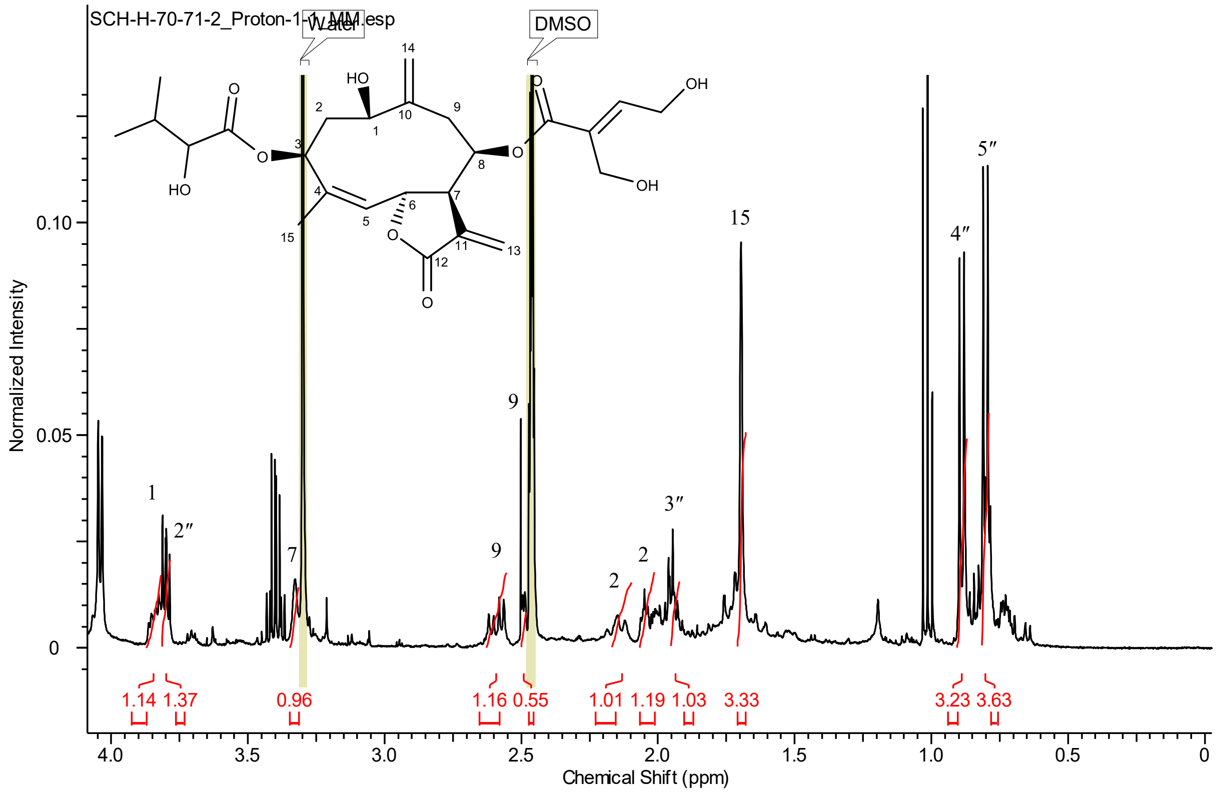

Figure S10. ^{1}H NMR spectrum of compound **2** in DMSO-d_6 in the range of 0–4.0 ppm

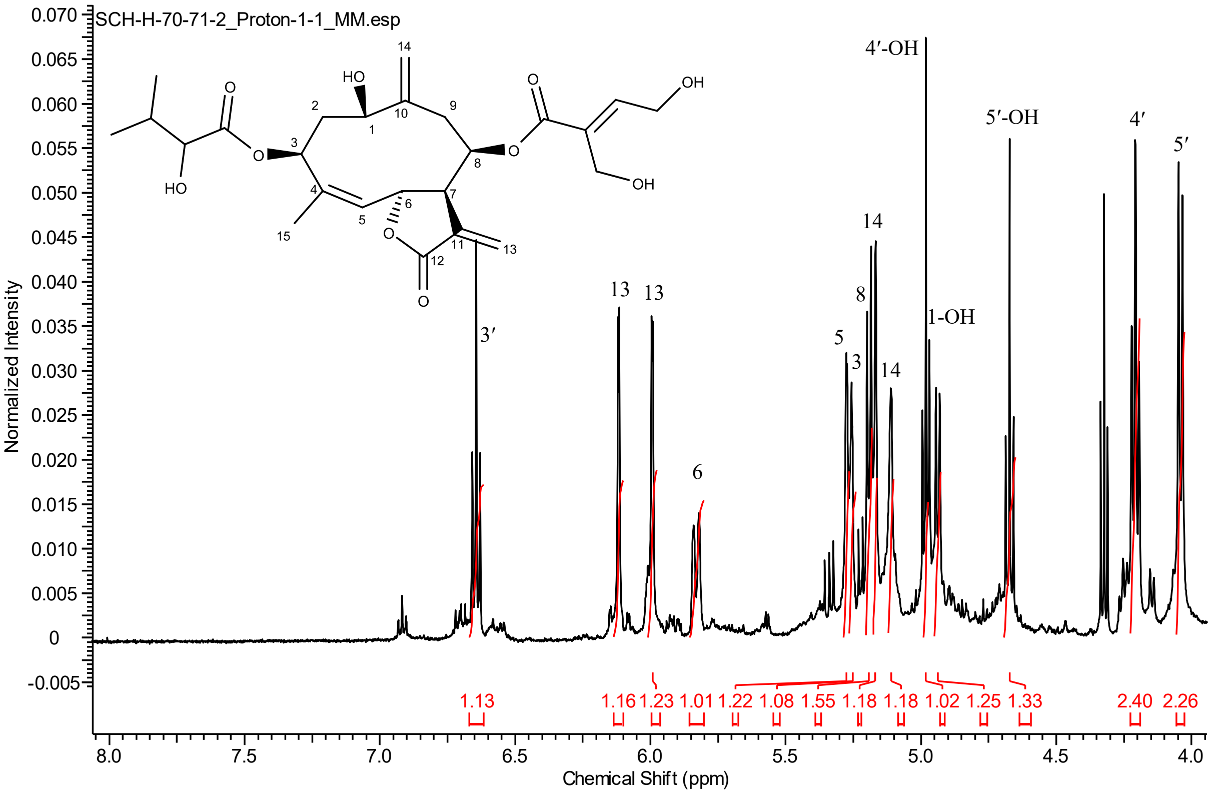

Figure S11. ^{1}H NMR spectrum of compound **2** in DMSO-*d*$_6$ in the range of 4.0–8.0 ppm

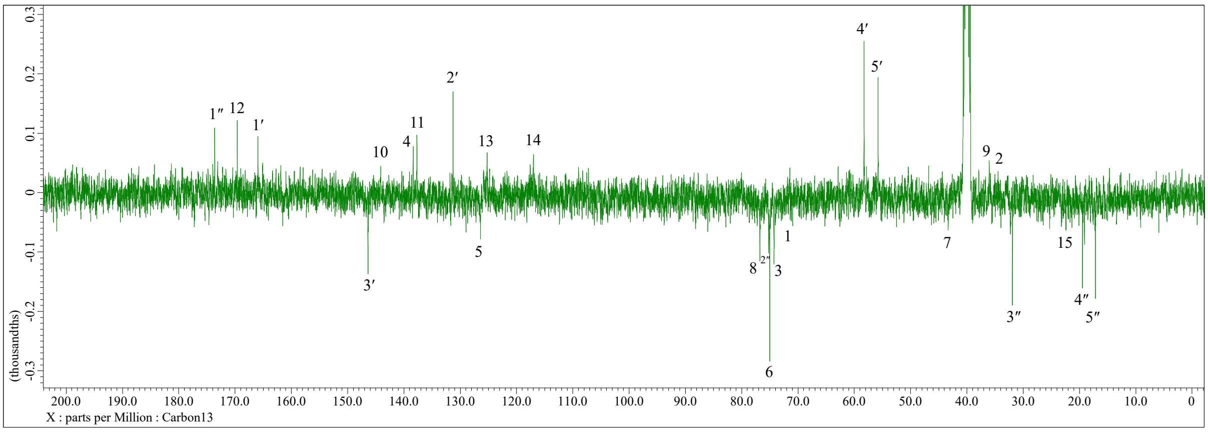

Figure S12. APT spectrum of compound **2** in DMSO-*d*$_6$

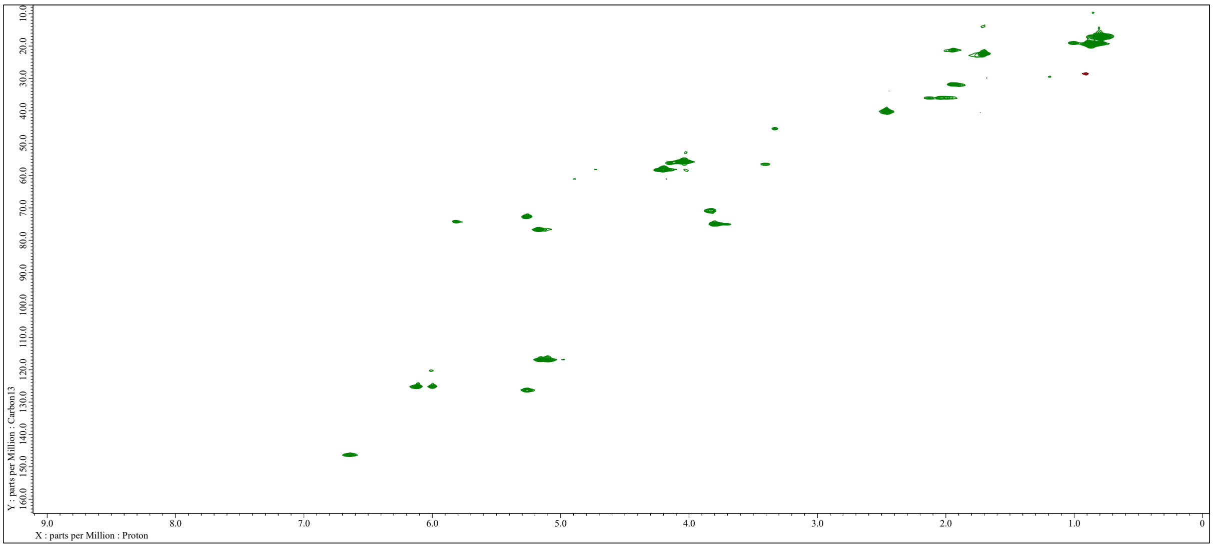

Figure S13. HSQC spectrum of compound **2** in DMSO-d_6

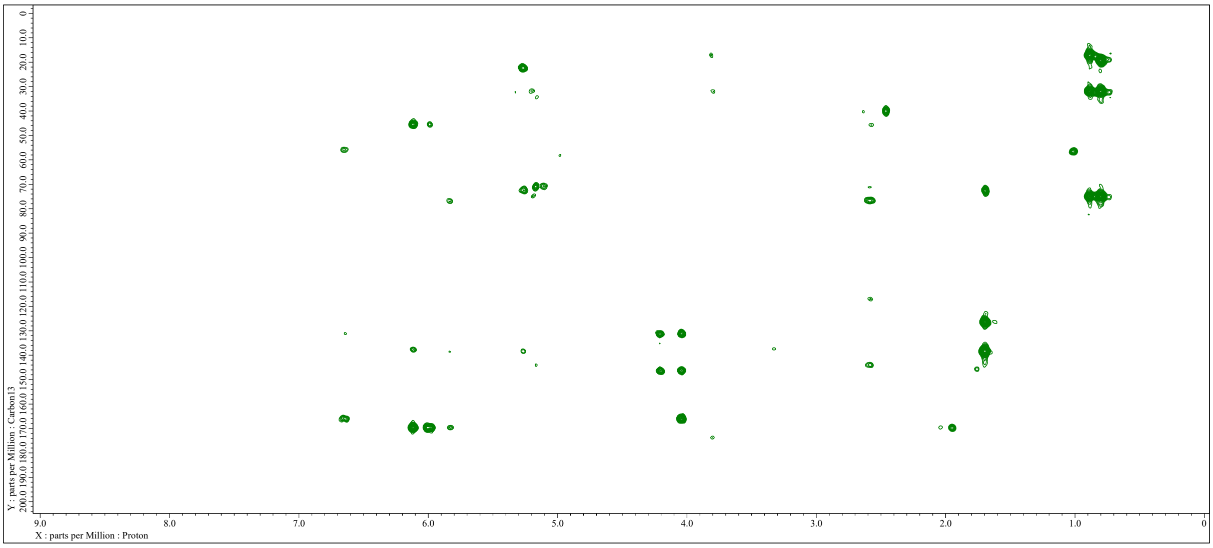

Figure S14. HMBC spectrum of compound **2** in DMSO-d_6

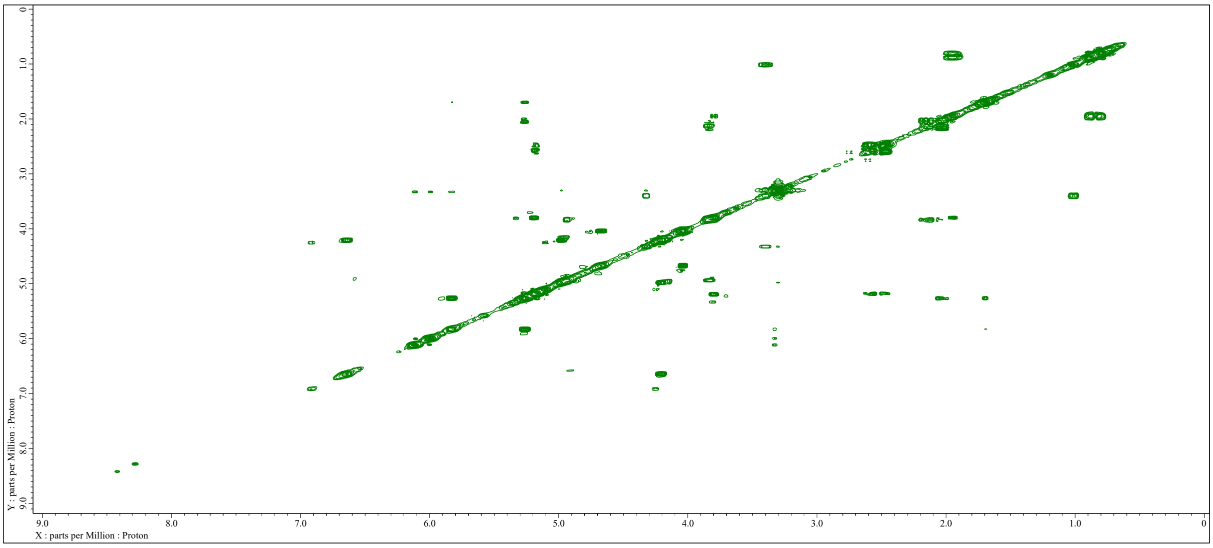

Figure S15. COSY spectrum of compound **2** in DMSO-d_6

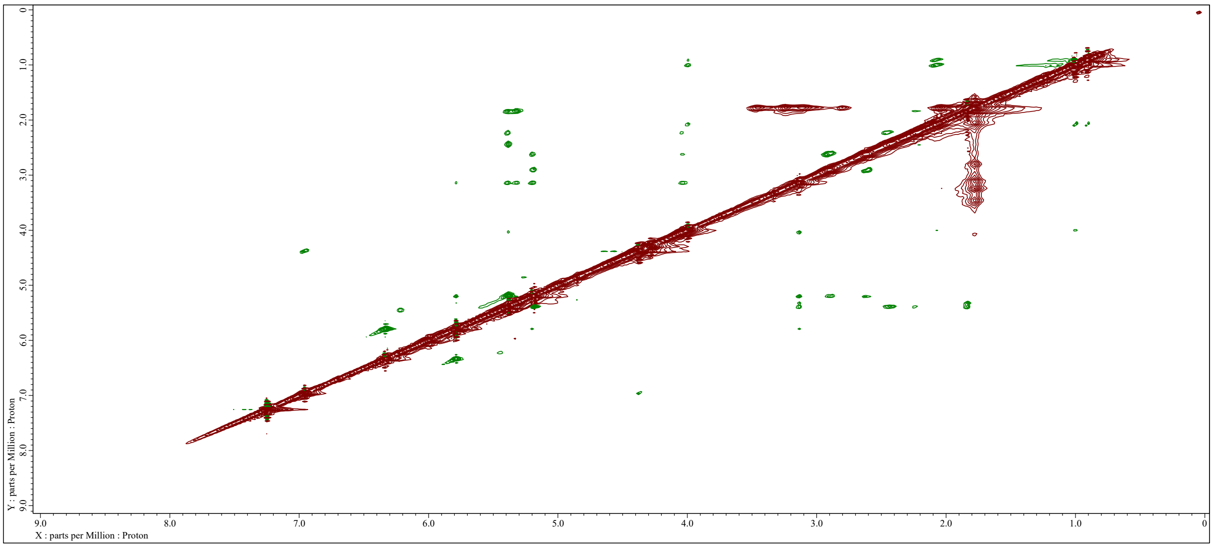

Figure S16. NOESY spectrum of compound **2** in $CDCl_3$

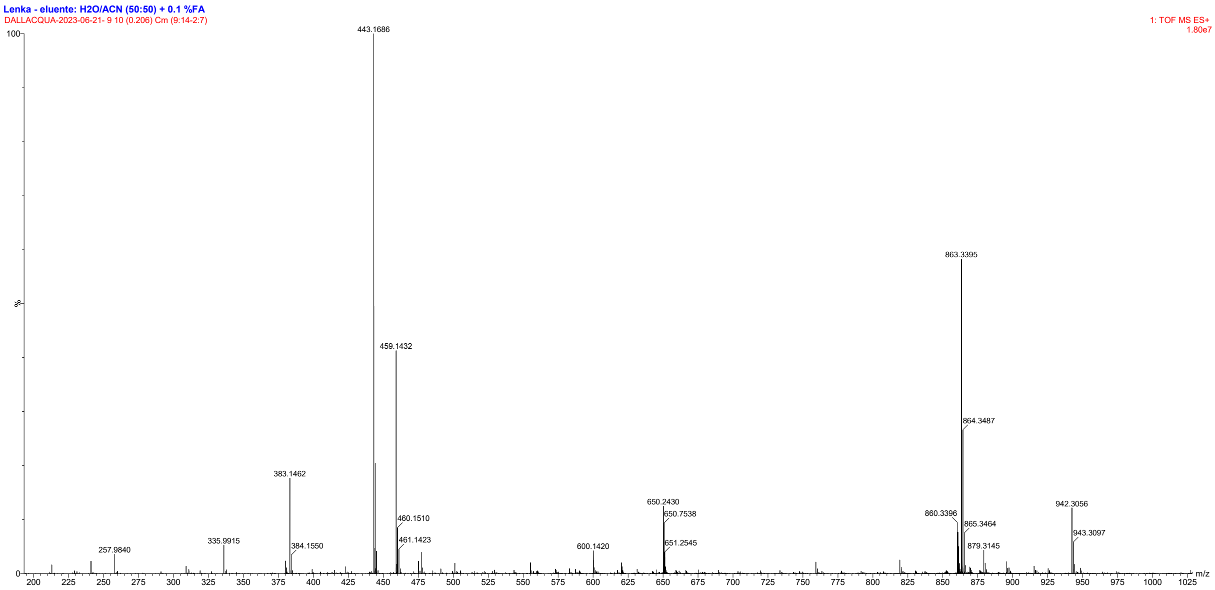

Figure S17. HRMS spectrum of compound **3** in positive mode

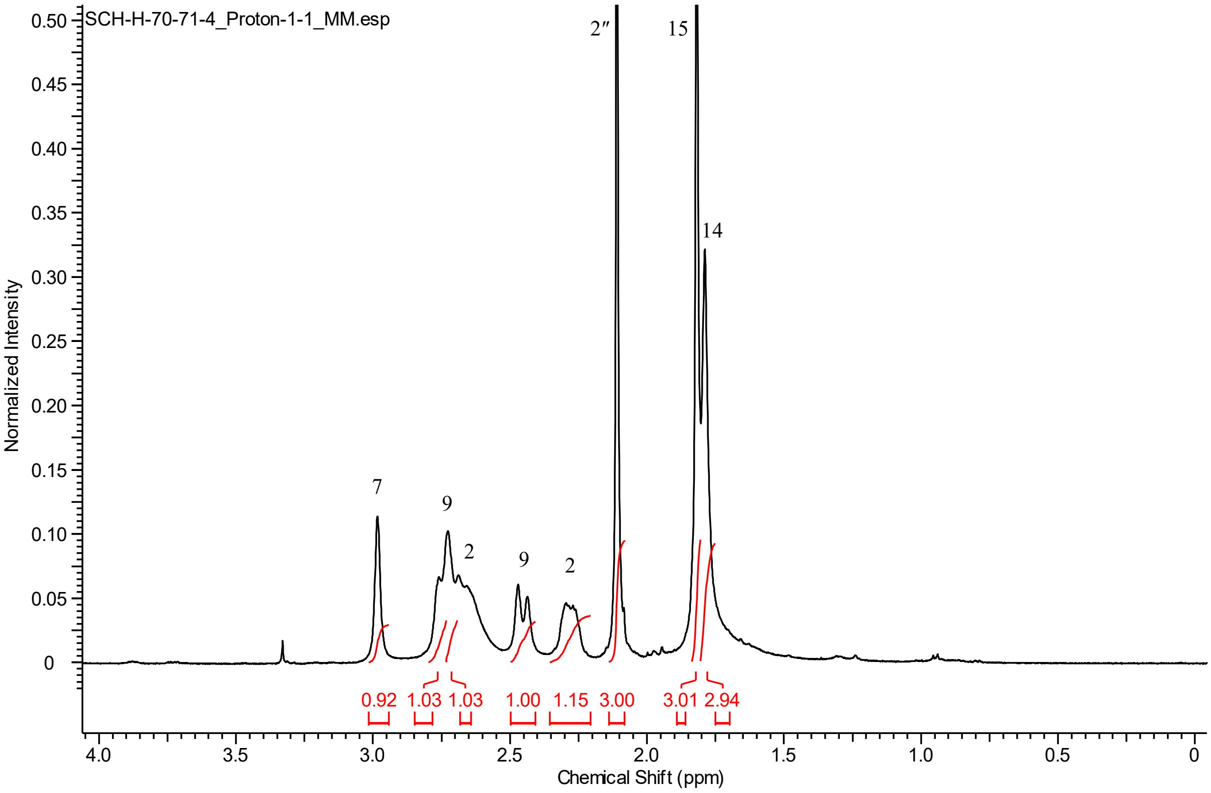

Figure S18. ^{1}H NMR spectrum of compound **3** in $CDCl_3$ in the range of 0–4.0 ppm

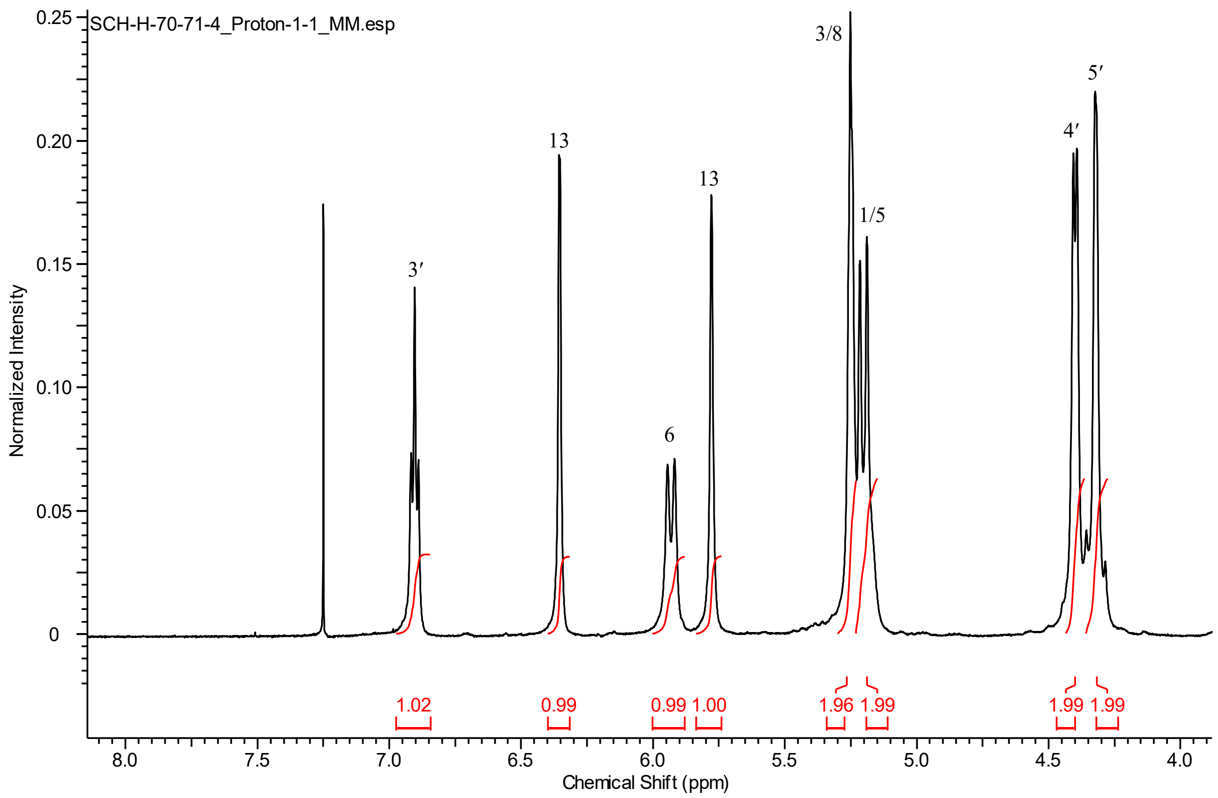

Figure S19. ^{1}H NMR spectrum of compound **3** in $CDCl_3$ in the range of 4.0–8.0 ppm

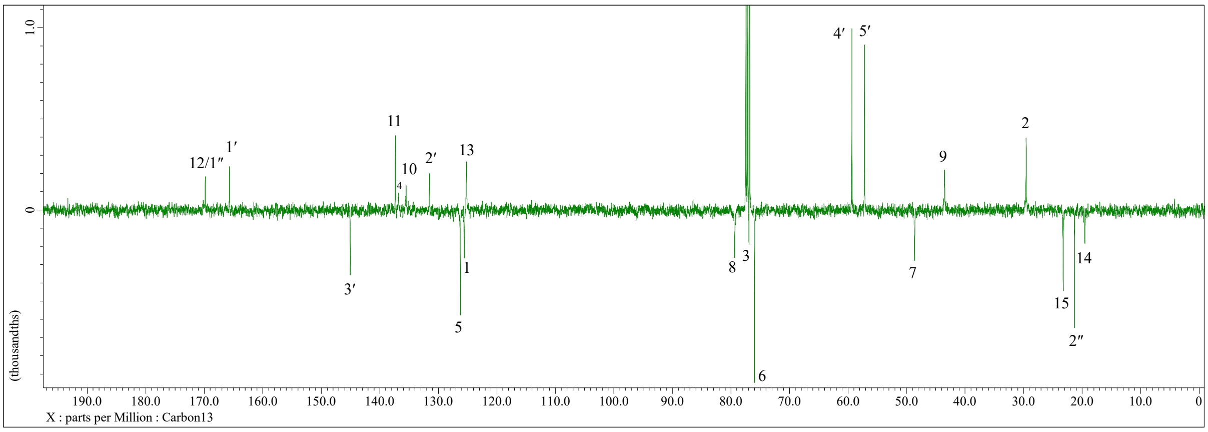

Figure S20. APT spectrum of compound **3** in $CDCl_3$

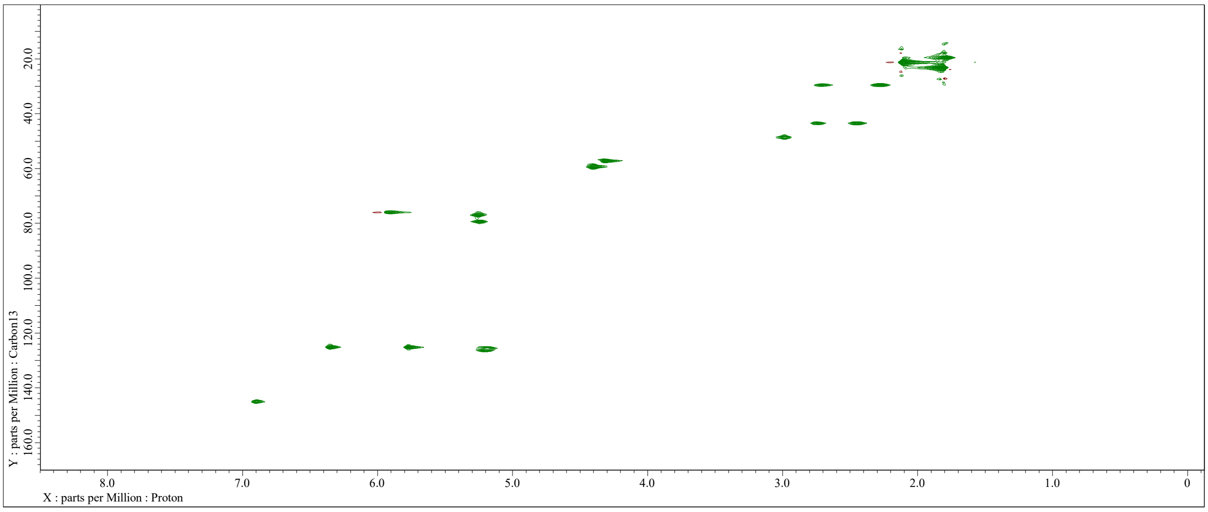

Figure S21. HSQC spectrum of compound **3** in $CDCl_3$

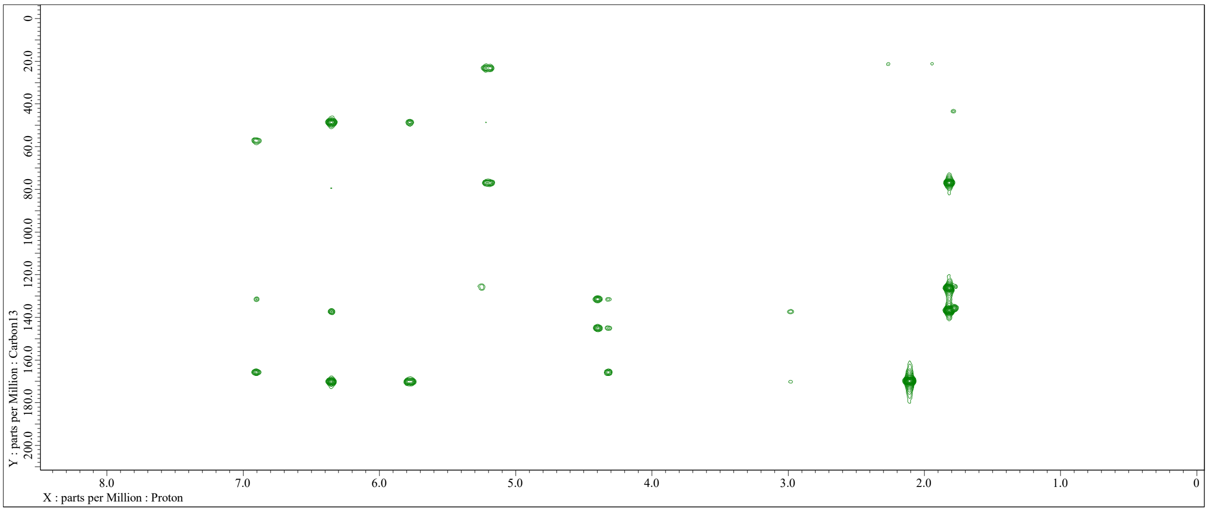

Figure S22. HMBC spectrum of compound **3** in $CDCl_3$

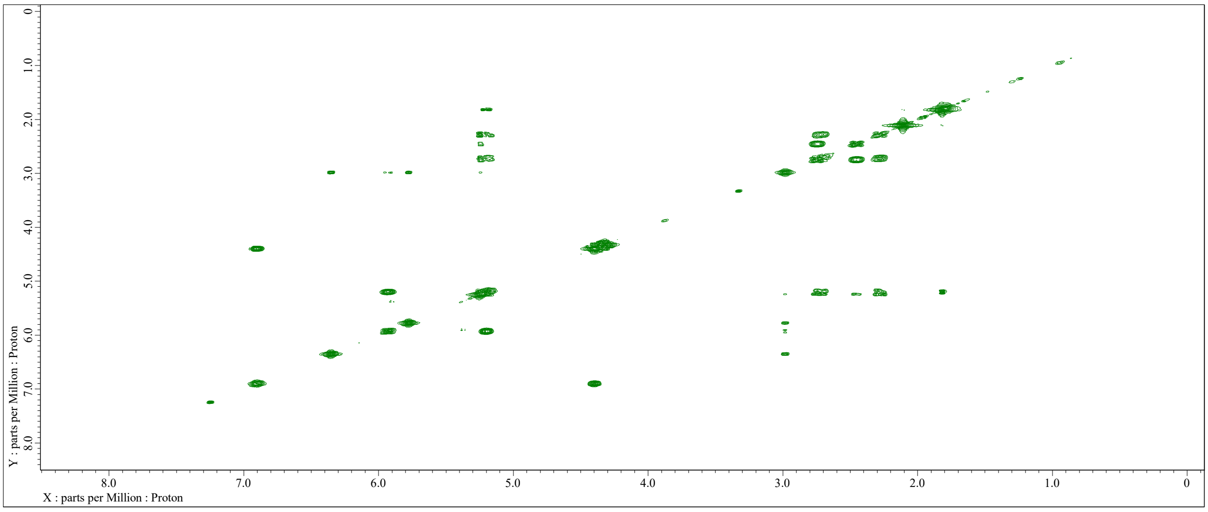

Figure S23. COSY spectrum of compound **3** in $CDCl_3$

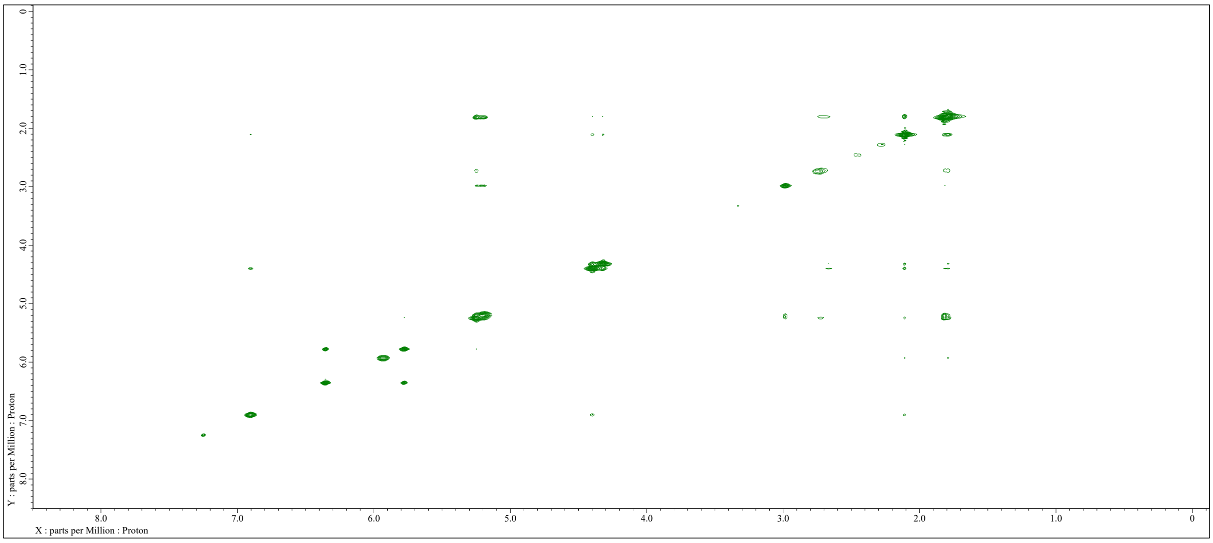

Figure S24. NOESY spectrum of compound **3** in $CDCl_3$

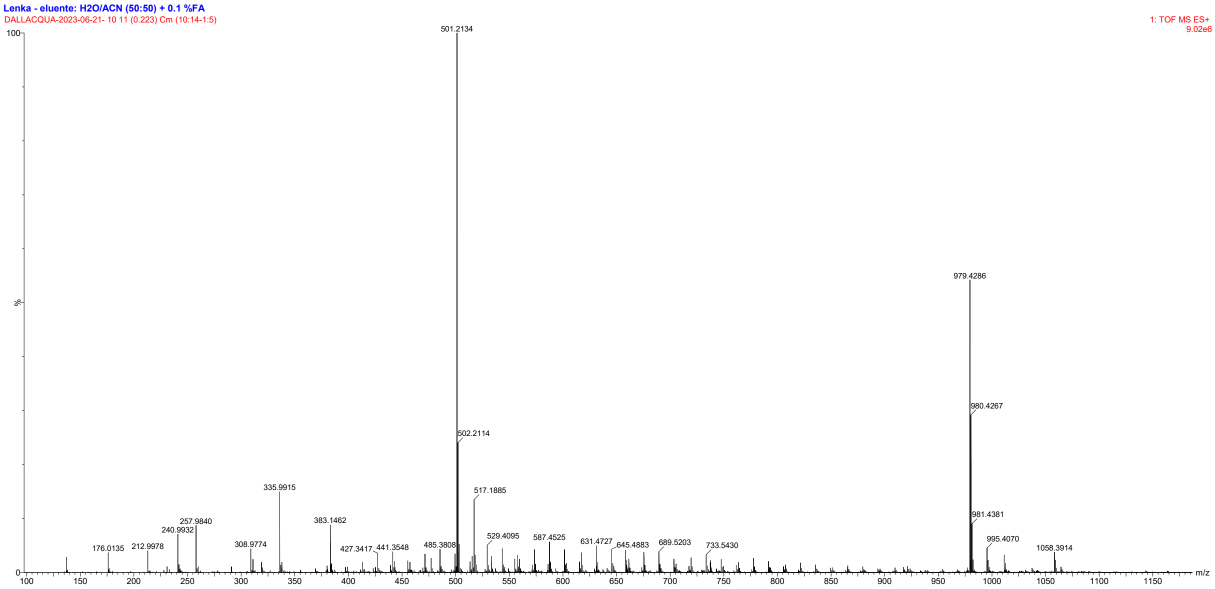

Figure S25. HRMS spectrum of compound **4** in positive mode

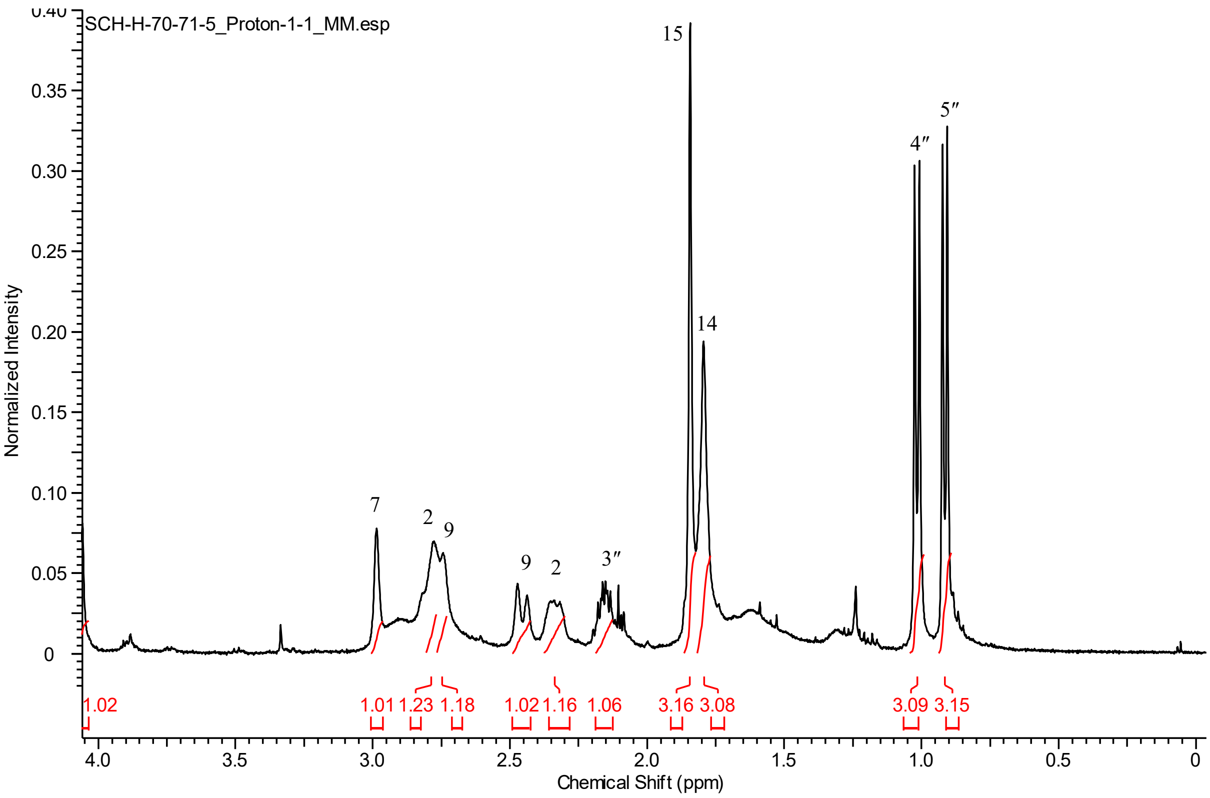

Figure S26. ^{1}H NMR spectrum of compound **4** in $CDCl_3$ in the range of 0–4.0 ppm

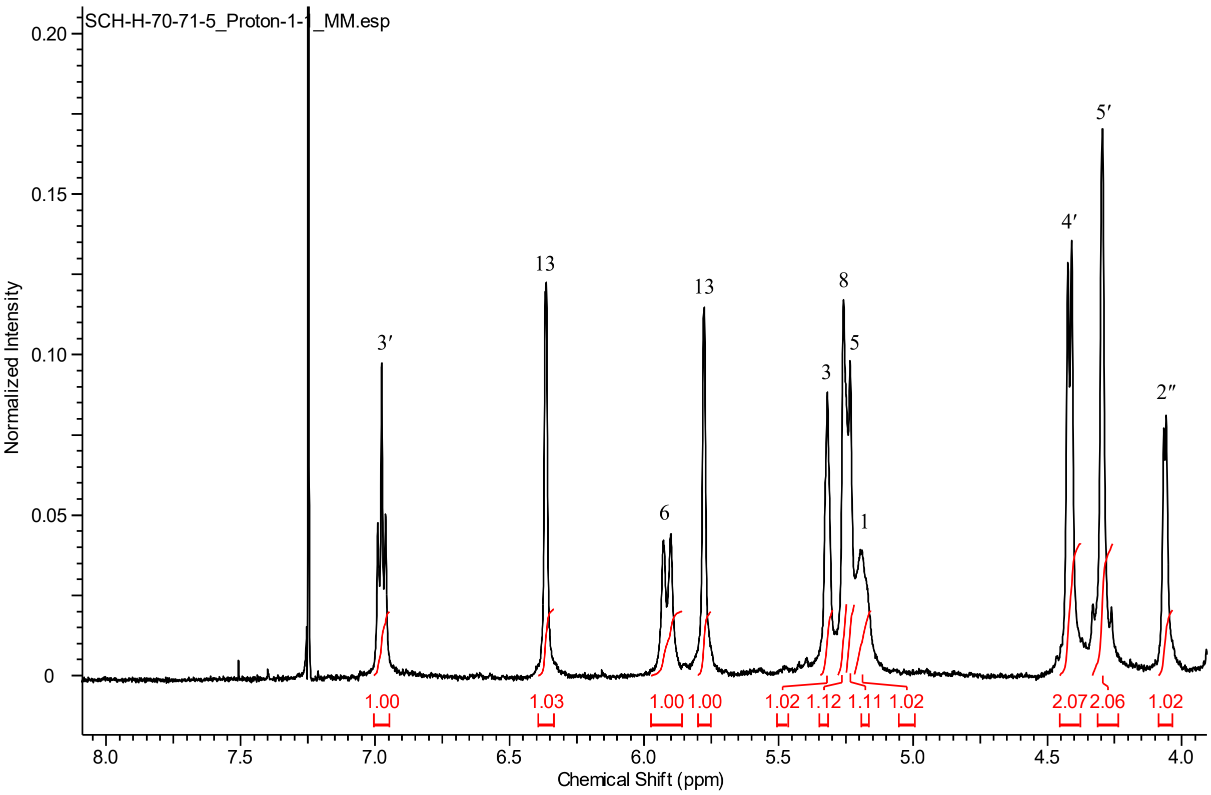

Figure S27. ^{1}H NMR spectrum of compound **4** in $CDCl_3$ in the range of 4.0–8.0 ppm

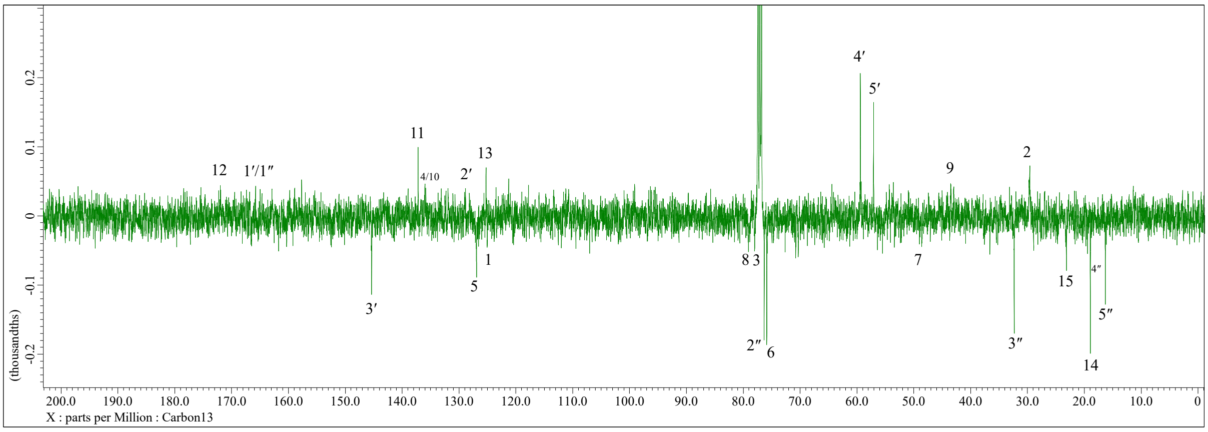

Figure S28. APT spectrum of compound **4** in $CDCl_3$

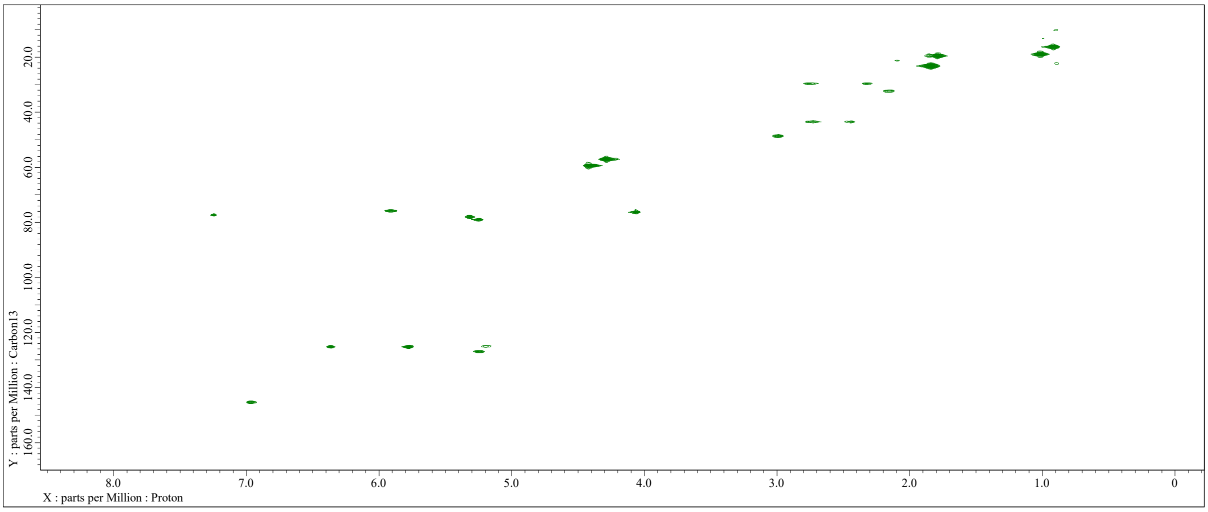

Figure S29. HSQC spectrum of compound **4** in $CDCl_3$

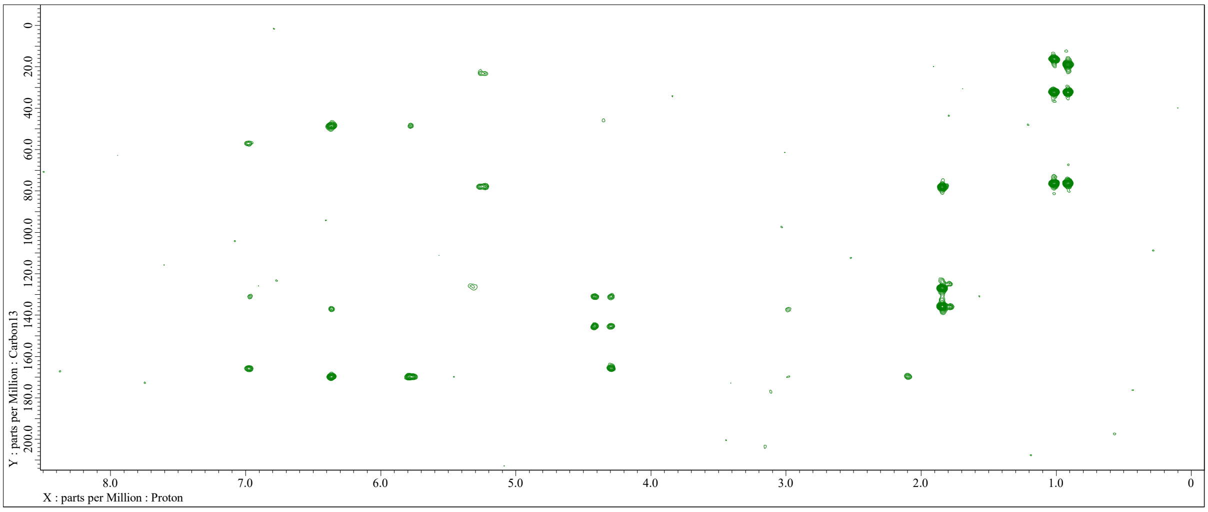

Figure S30. HMBC spectrum of compound **4** in $CDCl_3$

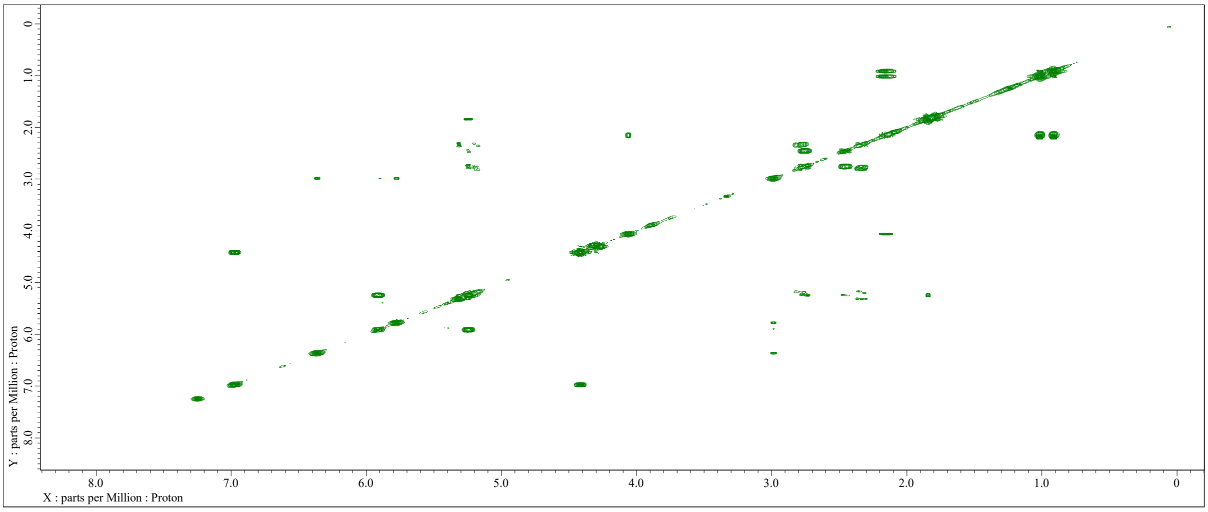

Figure S31. COSY spectrum of compound **4** in $CDCl_3$

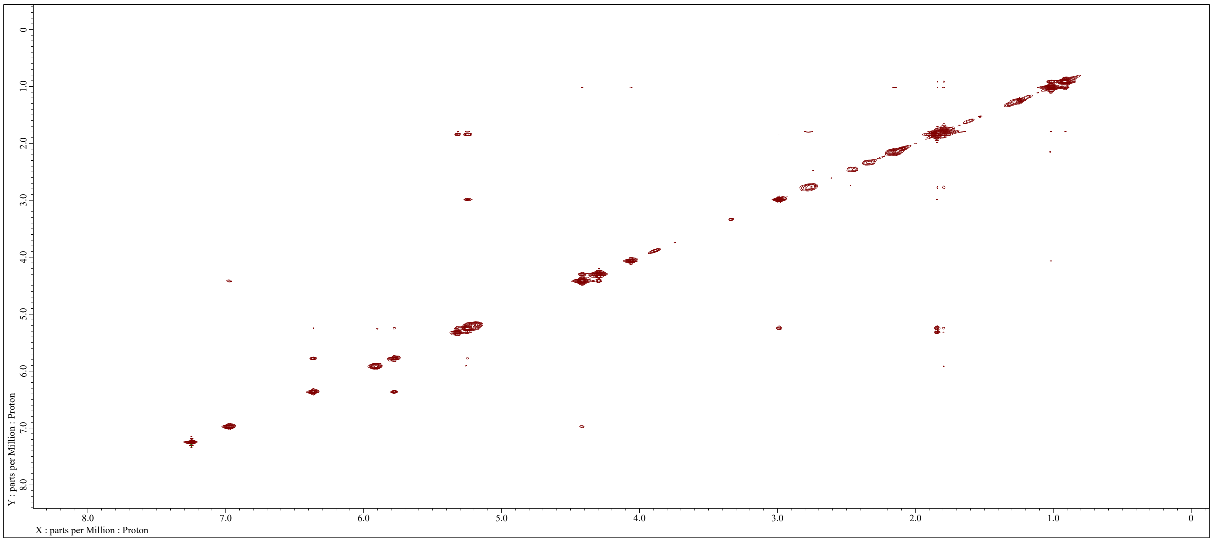

Figure S32. NOESY spectrum of compound **4** in $CDCl_3$

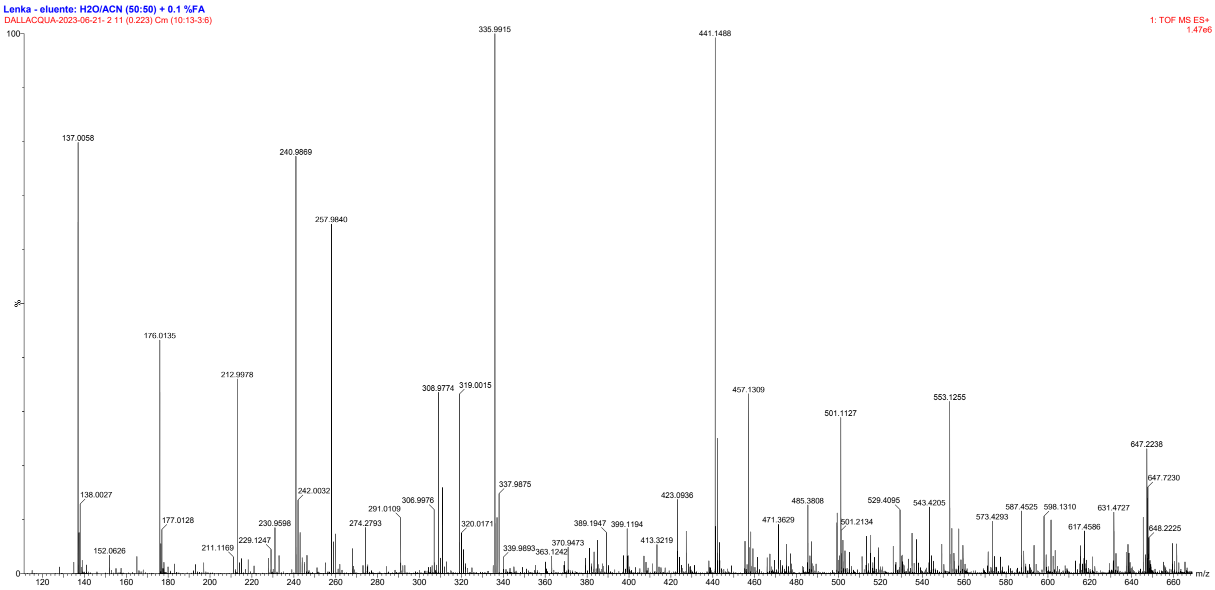

Figure S33. HRMS spectrum of compound **5** in positive mode

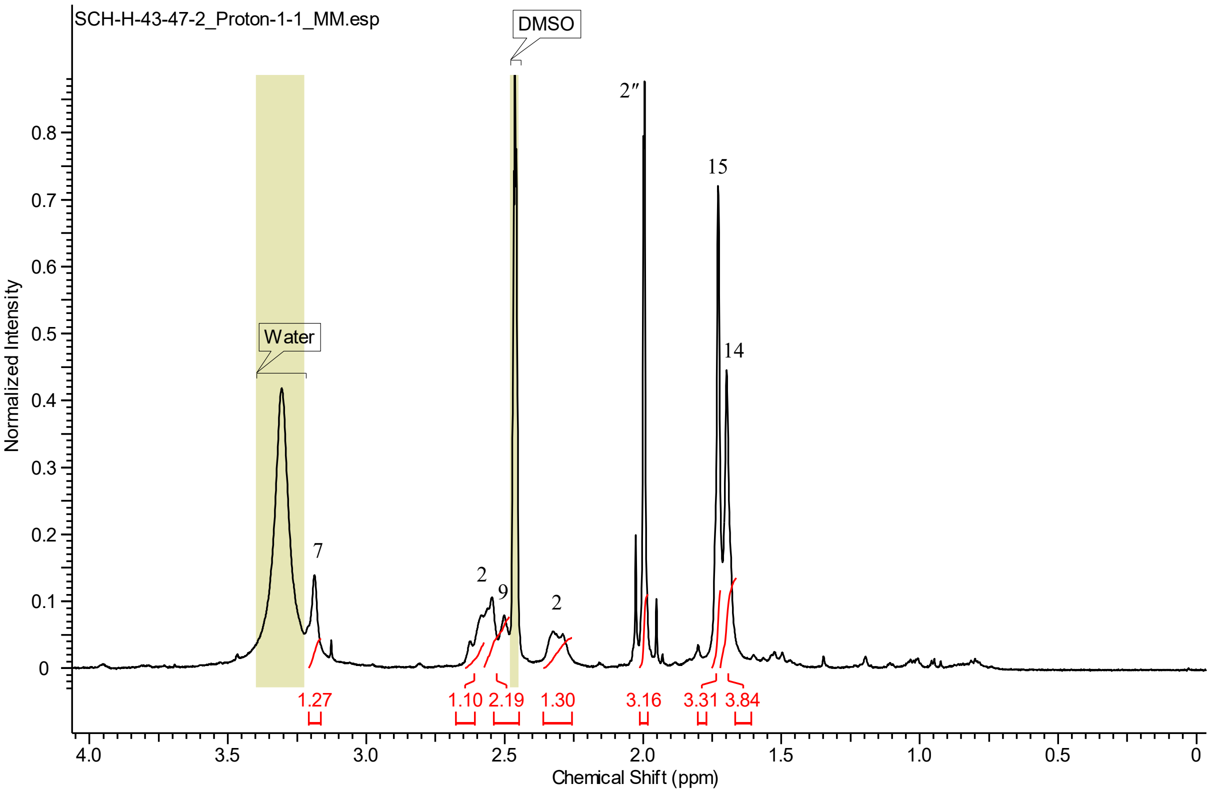

Figure S34. ^{1}H NMR spectrum of compound **5** in DMSO-d_6 in the range of 0–4.0 ppm

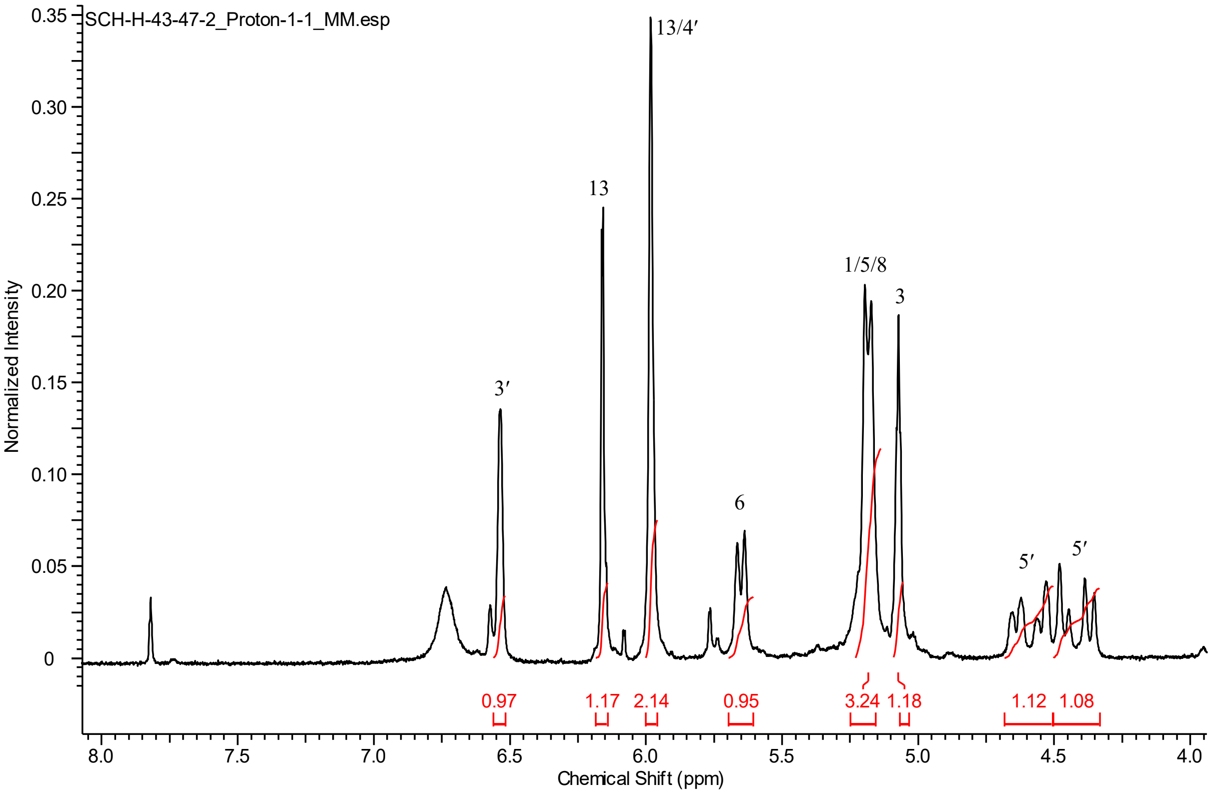

Figure S35. ^{1}H NMR spectrum of compound **5** in DMSO-d_6 in the range of 4.0–8.0 ppm

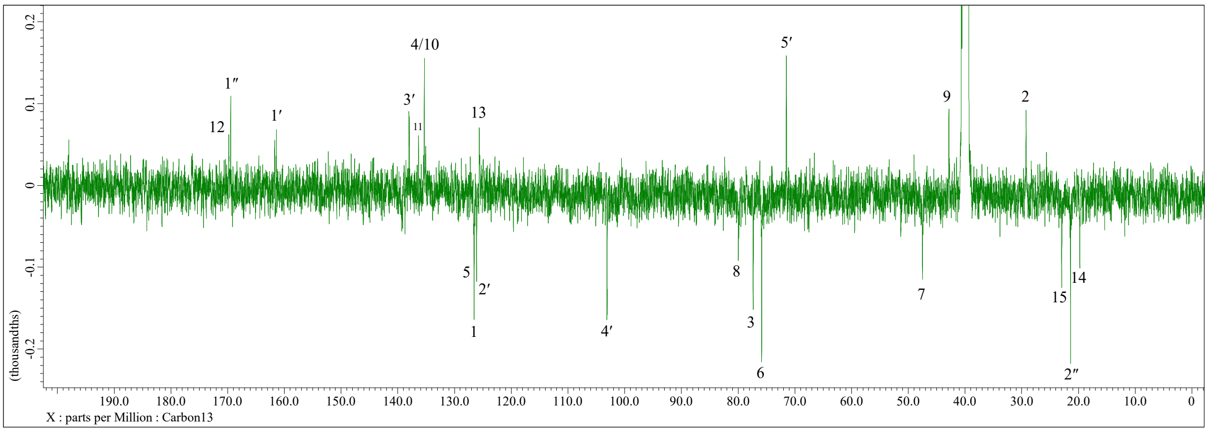

Figure S36. APT spectrum of compound **5** in DMSO-d_6

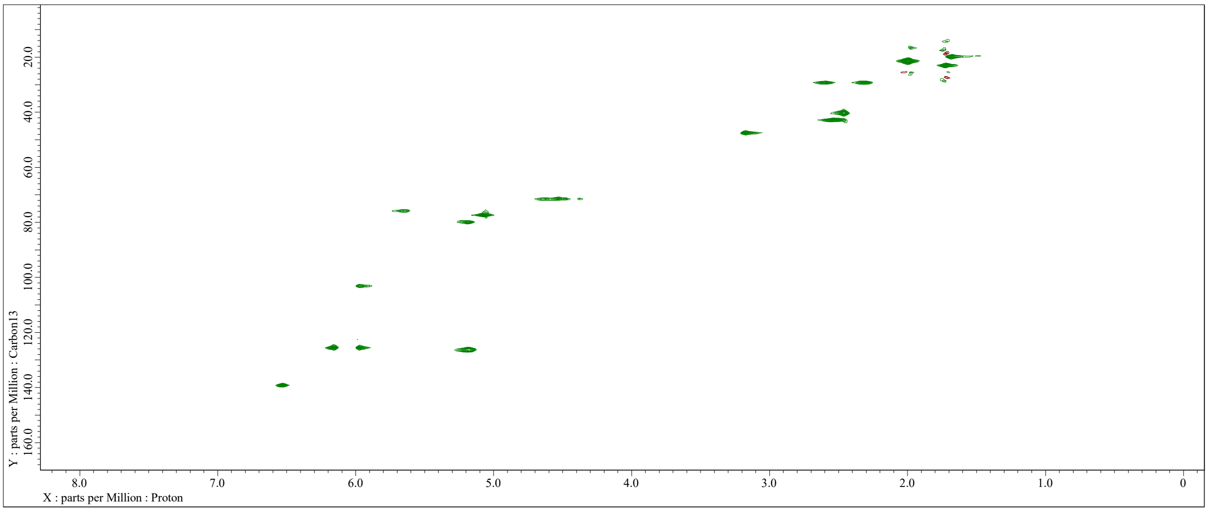

Figure S37. HSQC spectrum of compound **5** in DMSO-d_6

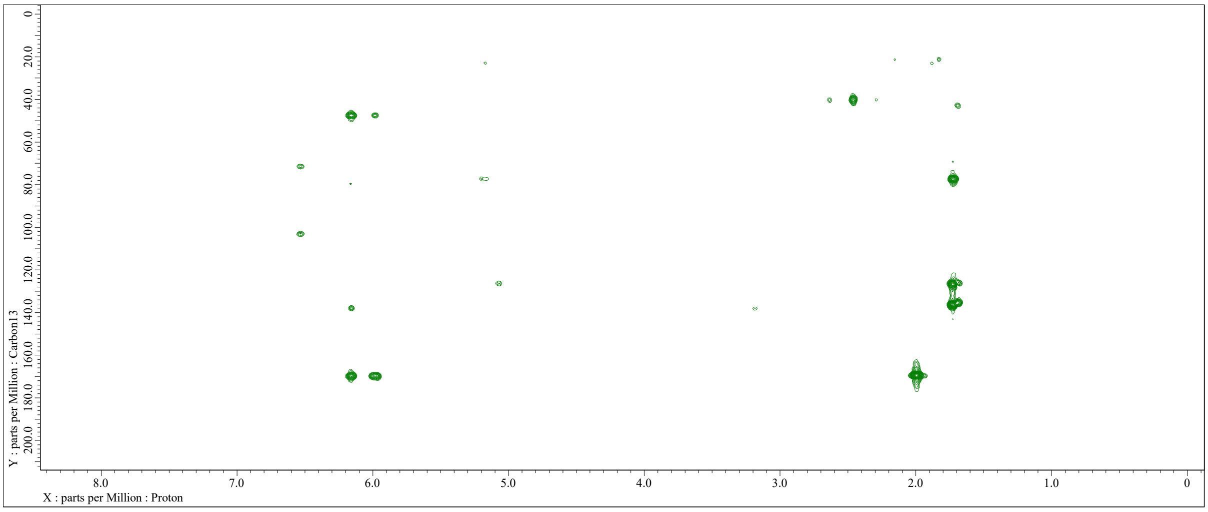

Figure S38. HMBC spectrum of compound **5** in DMSO-d_6

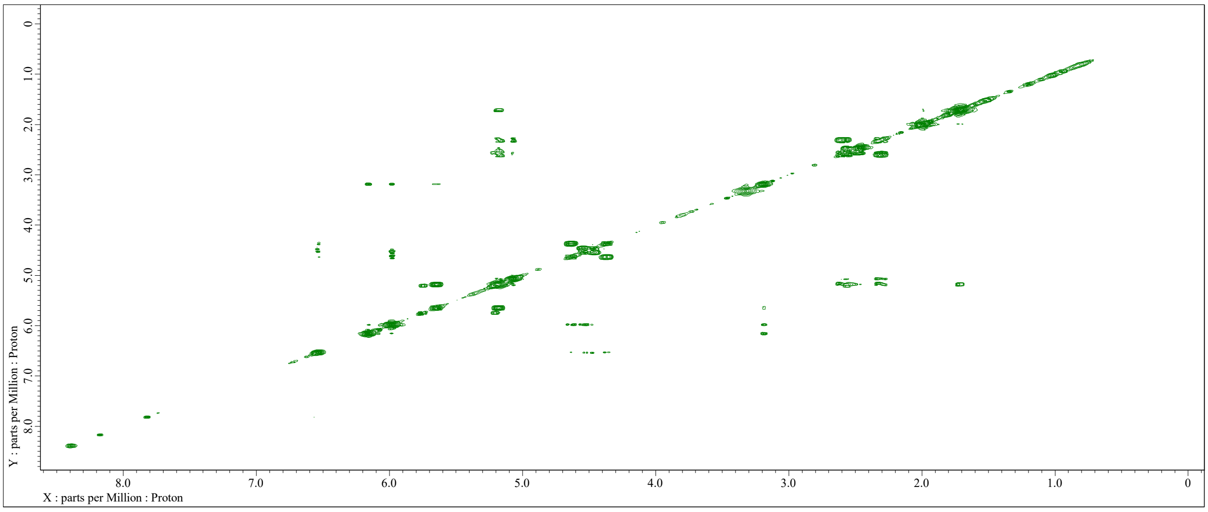

Figure S39. COSY spectrum of compound **5** in DMSO-d_6

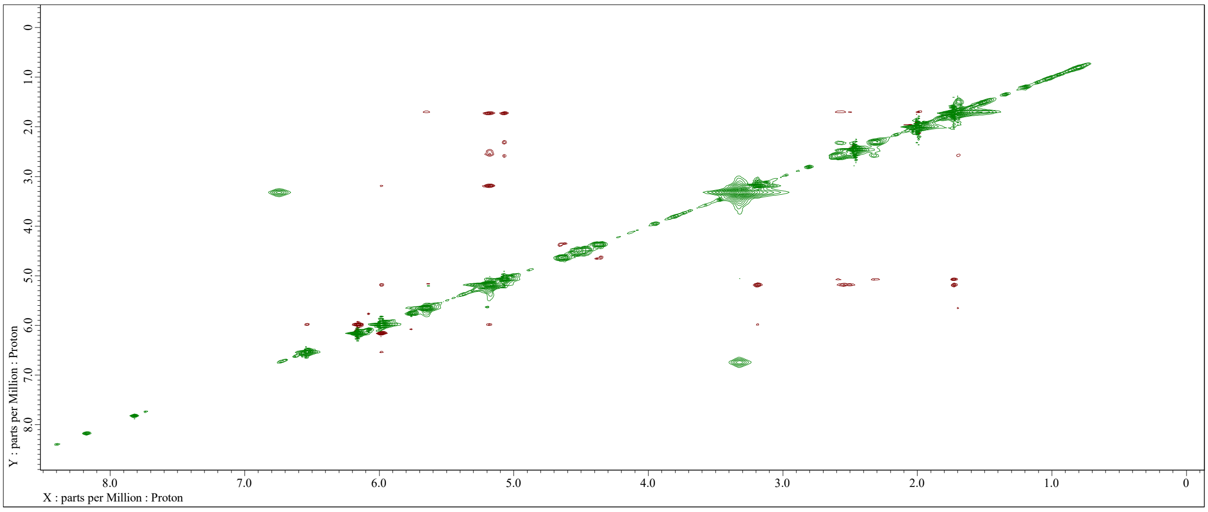

Figure S40. NOESY spectrum of compound **5** in DMSO-d_6

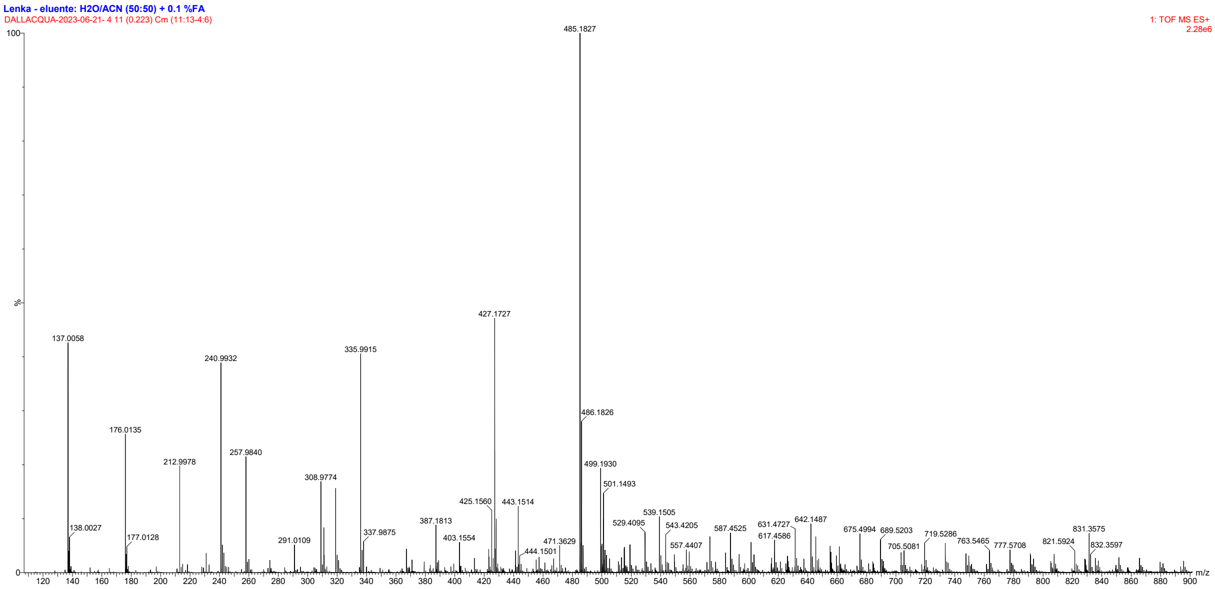

Figure S41. HRMS spectrum of compound **6** in positive mode

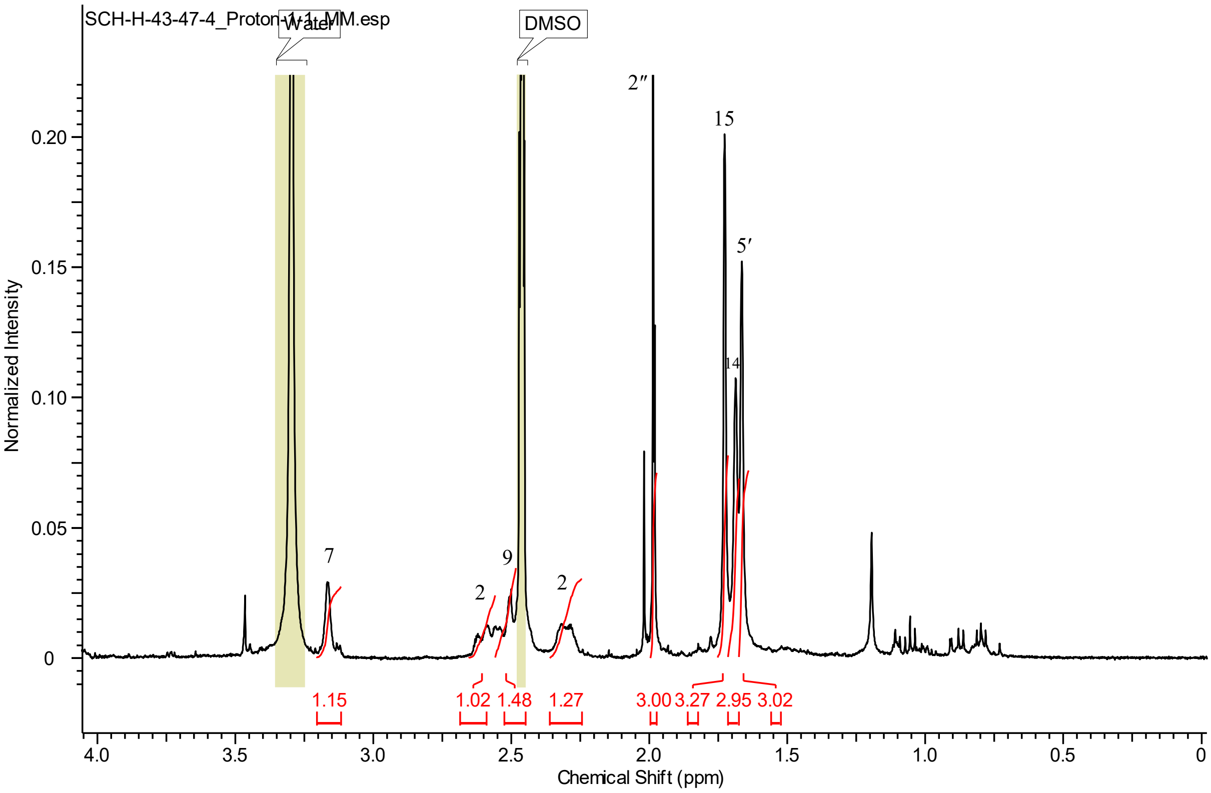

Figure S42. ^{1}H NMR spectrum of compound **6** in DMSO-d_6 in the range of 0–4.0 ppm

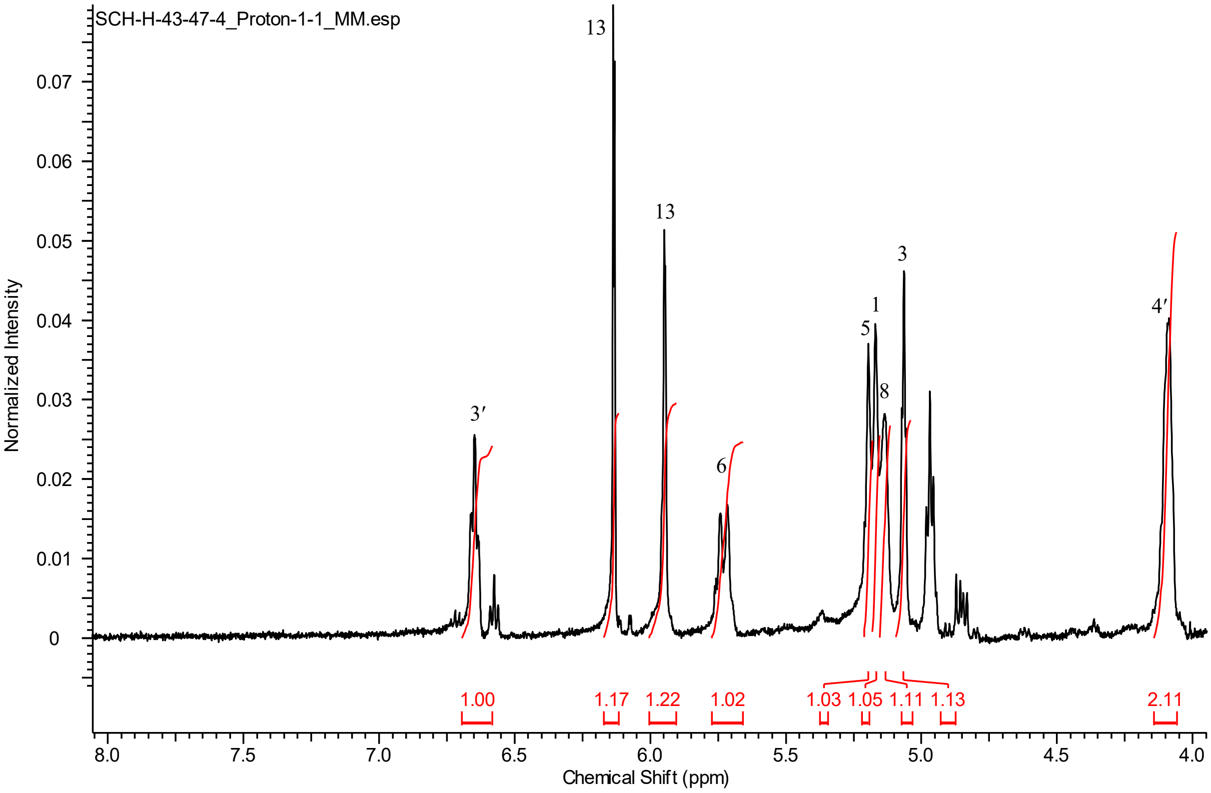

Figure S43. ^{1}H NMR spectrum of compound **6** in DMSO-d_6 in the range of 4.0–8.0 ppm

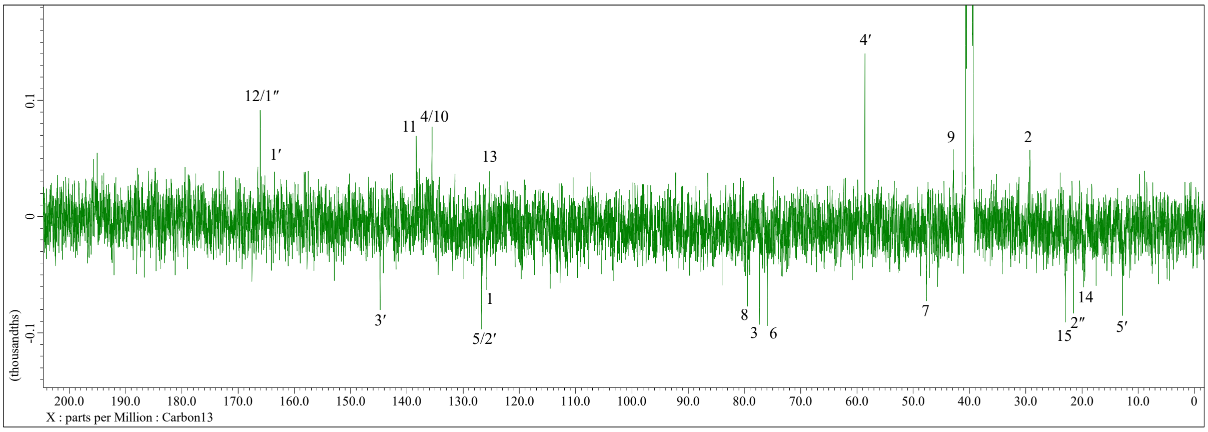

Figure S44. APT spectrum of compound **6** in DMSO-d_6

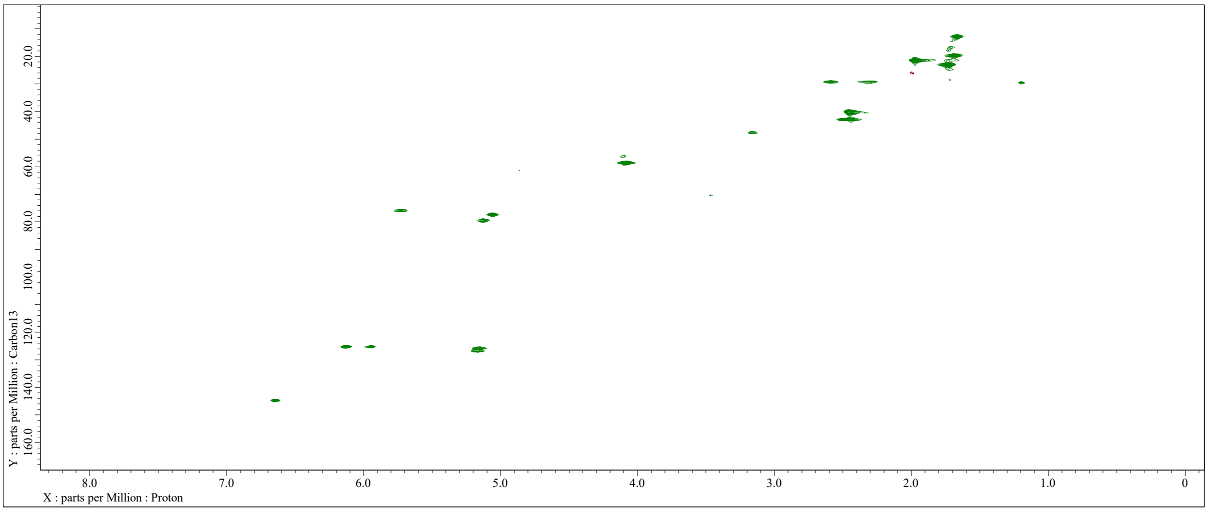

Figure S45. HSQC spectrum of compound **6** in DMSO-d_6

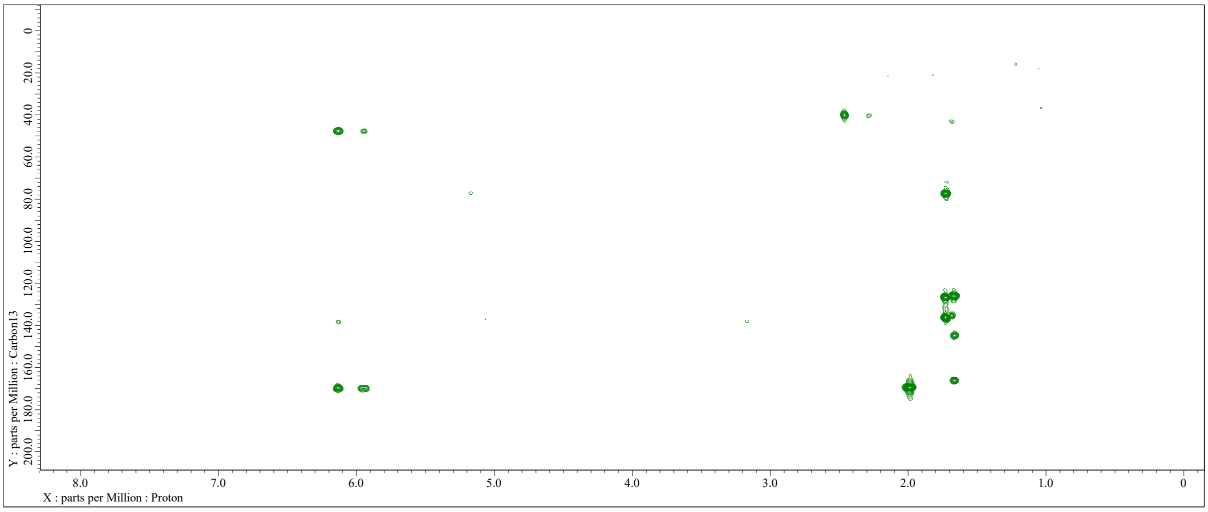

Figure S46. HMBC spectrum of compound **6** in DMSO-d_6

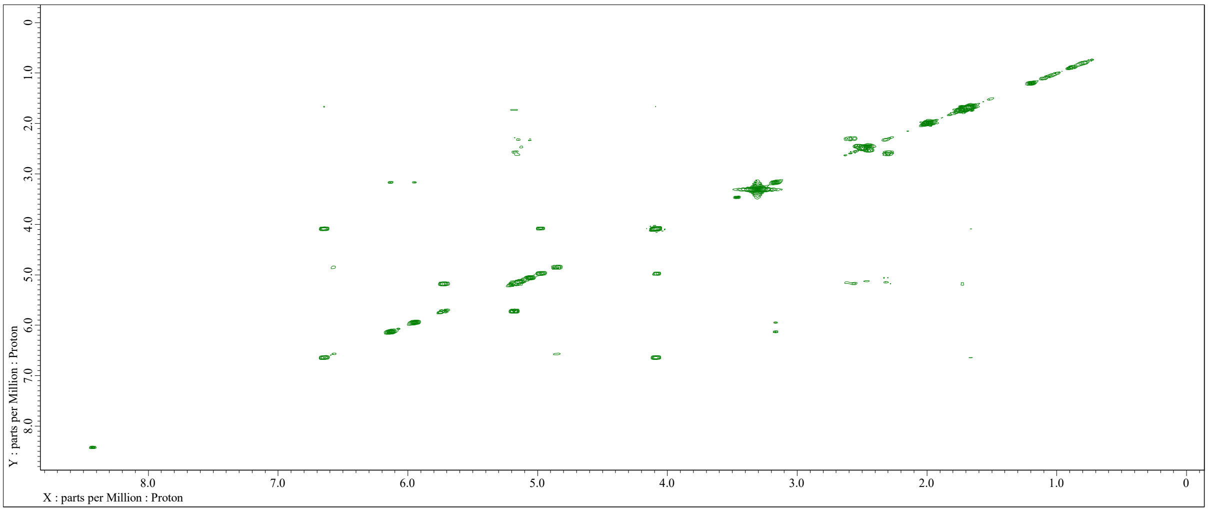

Figure S47. COSY spectrum of compound **6** in DMSO-d_6

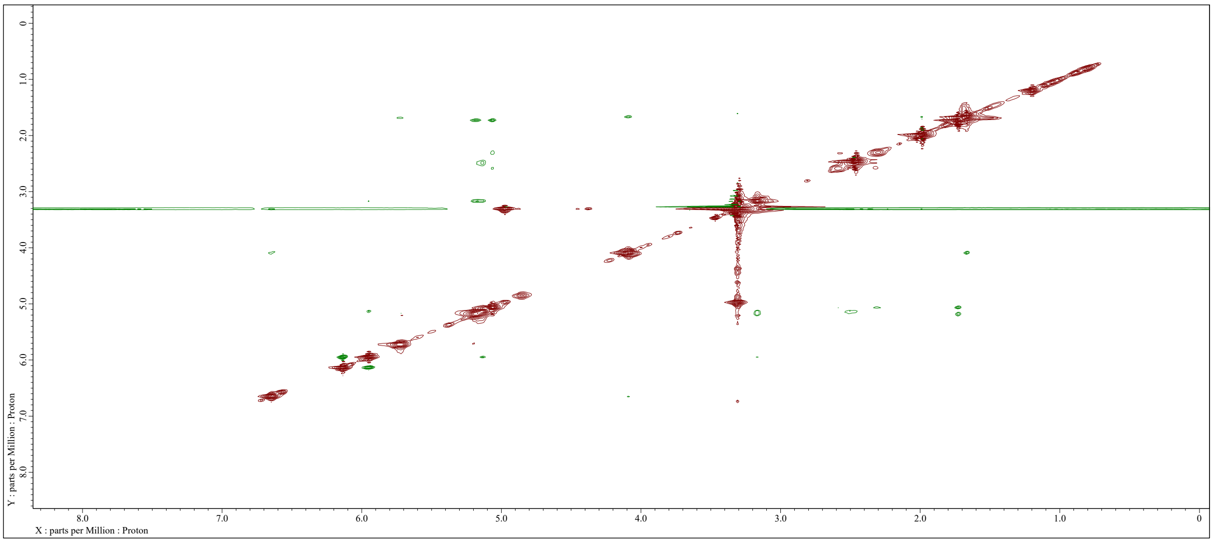

Figure S48. NOESY spectrum of compound **6** in DMSO-d_6
